# Eumelanin broadband absorption develops from aggregation-modulated chromophore interactions under structural and redox control

**DOI:** 10.1038/srep41532

**Published:** 2017-02-02

**Authors:** Raffaella Micillo, Lucia Panzella, Mariagrazia Iacomino, Giacomo Prampolini, Ivo Cacelli, Alessandro Ferretti, Orlando Crescenzi, Kenzo Koike, Alessandra Napolitano, Marco d’Ischia

**Affiliations:** 1 Department of Clinical Medicine and Surgery, University of Naples Federico II, I-80131 Naples, Italy; 2 Department of Chemical Sciences, University of Naples Federico II, I-80126 Naples, Italy; 3 Istituto di Chimica dei Composti Organometallici (ICCOM-CNR), Area della Ricerca, I-56124 Pisa, Italy; 4 Dipartimento di Chimica e Chimica Industriale, Università di Pisa, I-56124 Pisa, Italy; 5 Hair care Products Research Laboratories, Kao Corporation, Tokyo 131-8501, Japan

## Abstract

Eumelanins, the chief photoprotective pigments in man and mammals, owe their black color to an unusual broadband absorption spectrum whose origin is still a conundrum. Excitonic effects from the interplay of geometric order and disorder in 5,6-dihydroxyindole (DHI)-based oligomeric/polymeric structures play a central role, however the contributions of structural (scaffold-controlled) and redox (π-electron-controlled) disorder have remained uncharted. Herein, we report an integrated experimental-theoretical entry to eumelanin chromophore dynamics based on poly(vinyl alcohol)-controlled polymerization of a large set of 5,6-dihydroxyindoles and related dimers. The results a) uncover the impact of the structural scaffold on eumelanin optical properties, disproving the widespread assumption of a universal monotonic chromophore; b) delineate eumelanin chromophore buildup as a three-step dynamic process involving the rapid generation of oxidized oligomers, termed melanochromes (phase I), followed by a slow oxidant-independent band broadening (phase II) leading eventually to scattering (phase III); c) point to a slow reorganization-stabilization of melanochromes via intermolecular redox interactions as the main determinant of visible broadband absorption.

Eumelanins, black insoluble biopolymers found widespread in the animal kingdom, are commonly described as displaying broadband absorption spanning the entire visible range with monotonic wavelength dependence^1^,^2^. In human and mammalian skin and hair, eumelanins are produced by tyrosinase-catalyzed oxidation of tyrosine via polymerization of 5,6-dihydroxyindole (DHI) and 5,6-dihydroxyindole-2-carboxylic acid (DHICA)^3^. This latter process leads to the generation of complex mixtures of oligomeric species at various levels of oxidation and linked through diverse bonding patterns^4^. When followed spectrophotometrically, polymerization of DHI and DHICA proceeds with the development of broadband absorption profiles superposed to scattering contributions caused by eumelanin precipitation^1^. This process includes different levels of disorder, summarized in Supplementary Fig. 1, which account for the characteristic paramagnetic behavior^5^, water-dependent ionic-electronic conductivity^6^, and UV energy dissipation mechanisms^7^,^8^ of natural and synthetic eumelanins.

In the past decade, it was suggested that eumelanin broadband absorption properties could be explained in terms of the chemical disorder model^9^ which envisages the overlap of complex ensembles of chromophores spanning the entire UV-visible range. Studies of a water-soluble eumelanin model polymer produced from a glycated DHI derivative^10^ indicated however that the black chromophore reflects the *coexistence of oxidized and reduced moieties* within oligomer/polymer scaffolds. The discovery that poly(vinyl alcohol) (PVA) can prevent precipitation of growing eumelanin polymers allowed further investigation of eumelanin chromophore disentangled from scattering effects. The effect of PVA was attributed to the inhibition of aggregation based on dynamic light scattering evidence^11^. It was possible to conclude from those studies that eumelanin broadband absorption is not due simply to the overlap of static chromophore defined intrinsically by the conjugation length across the carbon frame^9^,^12^ but is also extrinsic in character resulting from aggregation-dependent intermolecular perturbations of the π-electron systems^13^. Extrinsic contributions appear to be dominant in DHICA polymers which exhibit repeated interruptions of interring conjugation due to lack of planar conformations (Fig. 1)^5^,^14^.

Very recently, several computational studies pointed out the importance of taking such extrinsic contribution into account^15^,^16^,^17^,^18^. It was suggested that excitonic coupling effects from the interplay of geometric order and disorder of aggregate structures broaden and enhance the visible absorption spectrum of eumelanin^15^. Delocalization of excitons over stacked eumelanin components would cause a general enhancement of absorption intensity explaining why eumelanin spectrum is monotonically increasing toward the higher-energy end, proportional to the cube of the absorption energy, and smoothly decaying to the lower-energy end. TD-DFT calculations confirmed the key role of π-stacking on the absorption spectrum in DHI pairs, giving evidence that such phenomenon holds even when different DHI redox or tautomeric species are stacked in pairs^17^. An *ab initio* wave-function study of the absorption behavior of DHI oligomers and of doubly and triply π-stacked species of these oligomers by the MP2 and the linear-response CC2 methods demonstrated the effect of an increasing degree of oligomerization of DHI and of an increasing degree of π-stacking of DHI oligomers on the onset of the absorption spectra and on the degree of red-shift toward the visible region of the spectrum^16^. These results reinforced the view that the optical properties of biological eumelanins can be simulated only by including catechol, semiquinone and quinone building blocks^19^. Although these models provide overall useful clues to inquire into eumelanin optical properties, they suffer from the lack of a solid background of structure-property relationships. A main gap concerns in particular the nature of the processes underlying the band broadening phenomenon and the main chemical functionalities involved in the intermolecular interactions underlying exciton coupling.

Herein we report, to the best of our knowledge, the broadest comparative investigation so far available on the formation and evolution of eumelanin chromophores, which relied on the availability in pure form of a collection of eumelanin precursors (Fig. 2) featuring different substituents, molecular size (monomers and dimers) and shape (isomeric dimers).

Aim of the study was to elucidate the relative role of intramolecular, intermolecular and dynamic contributions to eumelanin chromophore buildup, in order to assess the various levels of structural control over evolution of π-electron disorder and the main chemical mechanisms involved. By combining spectrophotometric experiments with computations, two crucial issues in eumelanin research were addressed, concerning the dynamics of band broadening during eumelanin chromophore buildup and the role of chemical disorder. In relation to chemical disorder, two major contributions were considered separately, namely *structural disorder,* reflecting the variety of σ-scaffolds, and *redox disorder,* as determined by catechol-semiquinone-quinone mixing and π-electron perturbations.

## Results

### The dynamics of eumelanin chromophore evolution

Preliminary experiments were directed to compare the generation and evolution of chromophores from the eumelanin precursors and derivatives in the presence and in the absence of 1% PVA using three different oxidizing systems, namely potassium ferricyanide, which operates by an outer sphere one-electron transfer mechanism, and sodium periodate, which induces two-electron oxidations via cyclic esters with catechols, both in phosphate buffer at pH 7.0, or ceric ammonium nitrate (CAN), a one-electron oxidant active at pH 3. Reactions were carried out on DHI as the parent substrate and the oxidant concentration was based on a two-electron oxidation stoichiometry relative to the monomer, formally corresponding to *o*-quinone formation. Under these conditions, complete substrate consumption was determined by HPLC analysis in all cases investigated.

Data showed similar chromophoric changes and evolution kinetics with ferricyanide and periodate, but less intense visible chromophores with CAN at pH 3.0 (for complete sets of UV data see Supplementary Fig. 2). Such differences were attributed to both a lower degree of polymerization and the inhibition of deprotonation processes in an acidic medium. On the basis of these results, ferricyanide was selected as the oxidant and oxidations were carried out with DHI at 50 μM concentration. Scattering contributions to the final spectra were quantitated by measuring the absorbance changes following filtration of the final oxidation mixtures through a 0.45 micron nylon membrane. The results indicated substantial, though not complete, inhibition of precipitate and scattering in the presence of PVA.

**Figure 3:**
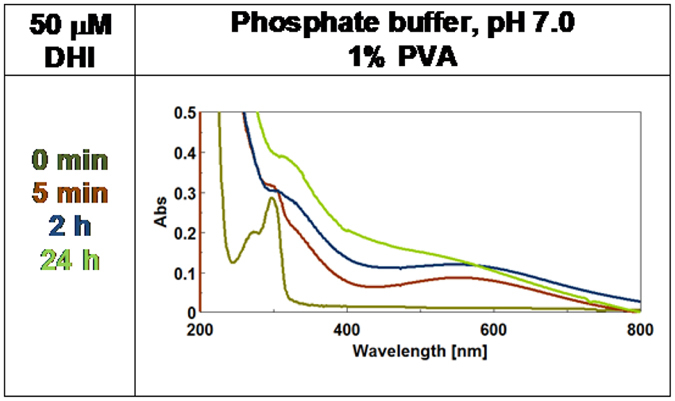
UV-Vis spectra of DHI oxidation over 24 hours reaction time in the presence of 1% PVA.

Supplementary Fig. 3 shows the time course of DHI oxidation over 24 hours reaction time. Data showed the generation of a broad visible chromophore around 560 nm, for which we propose the old name of melanochrome^20^. This attained maximum intensity within 5 min, was not affected by further addition of 2 molar amounts of oxidant (data not shown) and, following immediate filtration of the oxidation mixture through the membrane, was no more detectable in the spectrum of the filtrate (see Supplementary Fig. 3). On standing, the solution slowly darkened both in air and in an argon atmosphere due to broadening of the absorption band accompanied by extensive precipitation, as measured by filtration at 24 h. In the presence of 1% PVA (Fig. 3 and Supplementary Fig. 3), melanochrome formation proceeded in a similar manner though final precipitation of melanin was partly inhibited.

It can be concluded that eumelanin chromophore buildup by oxidation of DHI exhibits complex dynamics reflecting three main processes: phase I, i.e. the generation of intensely absorbing melanochrome, which is fast, depends on the availability of oxidant, and reflects mainly intrinsically-defined chromophores, e.g. oligomer species at various oxidation levels^21^; phase II, a band broadening process leading to solution darkening, which is slow and proceeds in an oxygen-independent manner, consistent with intermolecular chromophore perturbations accounting for extrinsically determined absorptions; and phase III, the onset of scattering due to eumelanin precipitation.

Contributions from phases II and III are almost superposed in the absence of PVA but can be partly disentangled in the presence of PVA, which markedly delays the contribution of scattering. On visual inspection, these three phases correspond to the development of a purple-violet or bluish coloration more evident in the presence of PVA, followed by gradual darkening and eventually deposition of a black solid (Supplementary Fig. 4). The effect of aggregation was apparent by comparing mixture colors at 5 min oxidation in the presence and in the absence of PVA, and mostly by the effect of filtration. In the absence of PVA, a complete removal of the melanin pigment formed was observed following filtration of the oxidation mixture (Supplementary Fig. 4)

**Figure 4:**
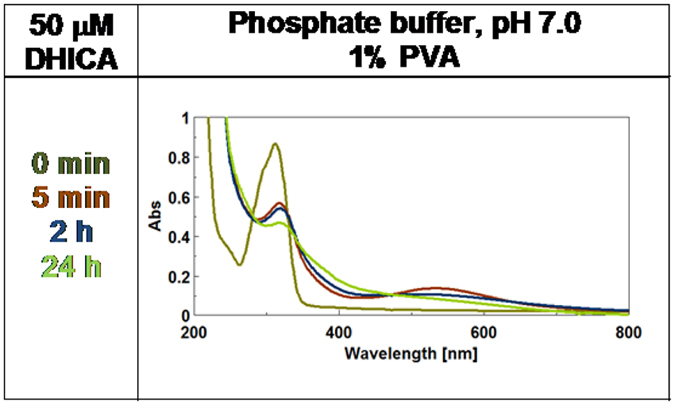
UV-Vis spectra of DHICA oxidation over 24 hours reaction time in the presence 1% PVA.

To probe the effect of ring substituents on the dynamics of DHI polymerization, the oxidation of the N-methyl derivative of DHI (N-MeDHI) and DHICA was next compared. Similar chromophore evolution dynamics were observed for N-MeDHI oxidation compared to DHI, suggesting negligible effects of the N-methyl group (Supplementary Figs 5 and 6). However a markedly different spectrophotometric course was determined in the case of DHICA (Fig. 4 and Supplementary Fig. 7). In this latter case, a deep violet chromophore broadly centered between 500–550 nm was generated both in the presence and in the absence of PVA, accompanied by a well-defined UV absorbing band around 330 nm, slightly shifted relative to the monomer maximum (312 nm). Interestingly, the intensity at 5 min and 2 h of the visible band centered around 530 nm was enhanced in the presence of PVA suggesting a delayed decay of melanochrome components under conditions of inhibited aggregation.

Visual inspection of the reaction mixtures from DHICA in the presence and in the absence of PVA revealed again the important role of aggregation in color development. An important difference with respect to DHI oxidation was the lighter coloration of the final melanin both in the presence and in the absence of PVA. The role of PVA as retarder of band broadening processes was also well apparent after 24 h (Supplementary Fig. 8).

### The role of structural disorder

**Figure 5:**
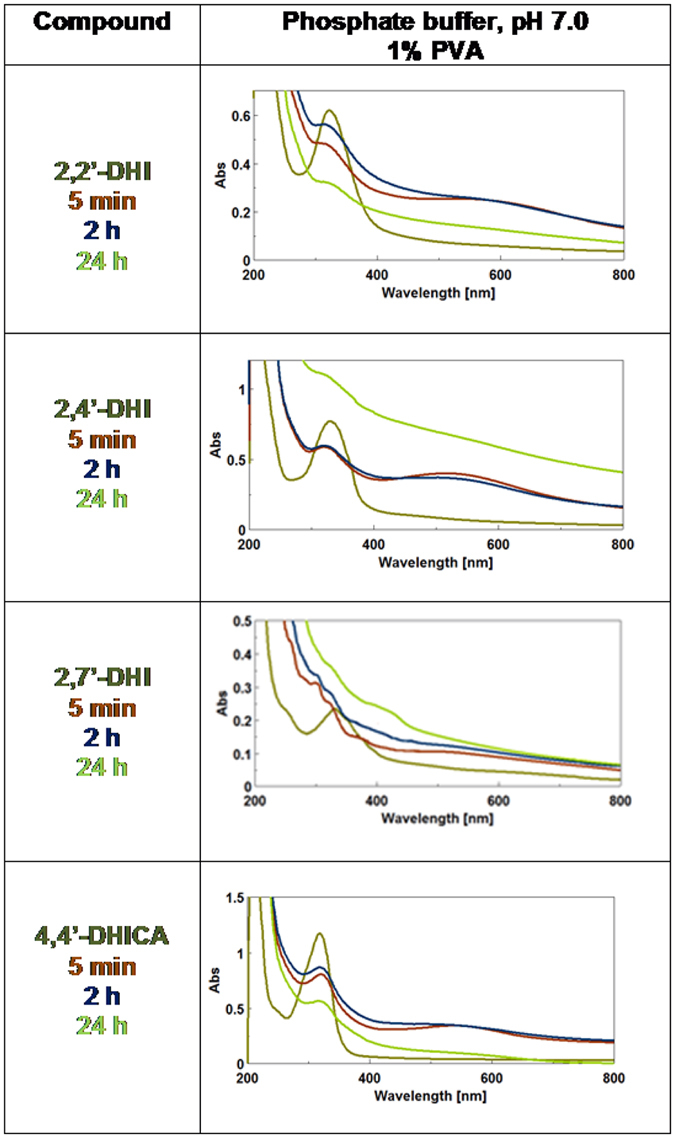
Generation of chromophores from DHI and DHICA dimers in the presence of 1% PVA. The oxidant concentration was based on a two-electron oxidation stoichiometry relative to the dimer.

Structural disorder encompasses both the number of gradational oligomer species produced in the polymerization process (molecular weight dispersion) and the variety of scaffolds produced within each oligomer population (molecular diversity). The effect of these two components of structural disorder on eumelanin chromophore buildup was specifically addressed by comparing the spectrophotometric course of the oxidation of dimers relative to the corresponding monomers. The rationale was that oxidation of dimers would reasonably produce a lower molecular weight dispersion (only even-numbered oligomers) and a lower variety of scaffolds with respect to the monomer, due to the inherent constraint posed by the interring bond. In addition, comparison of the chromophores produced from the isomeric 2,4′-, 2,7′- and 2,2′-dimers of DHI would allow to disclose possible effects of scaffold-controlled reactivity. The results, reported in Fig. 5, showed marked differences among the initially generated melanochrome chromophores of the various DHI dimers, both in the presence and in the absence of PVA (see also Supplementary Figs 9–11), which persisted in part in the final eumelanin polymers as determined after 24 h.

Apparently faster melanochrome evolution was observed for the 2,2′-dimer in the absence of PVA, as indicated by the almost overlapping traces recorded at 5 min, 2 h and 24 h (Supplementary Fig. 9). It can be suggested that it is the way the indole units are covalently bound (2,2′, 2,4′ or 2,7′), rather than molecular weight dispersion, that affects eumelanin chromophore formation. This influence is relevant in phase I, i.e. intrinsic chromophores, but is partly obscured during intermolecular evolution processes in phase II due to the partial homogenization of chromophores with broadening and color darkening. The oxidation course of 4,4′-DHICA (see also Supplementary Fig. 12) did not substantially differ from that of DHICA, though melanochrome evolution seems to be faster.

**Figure 6:**
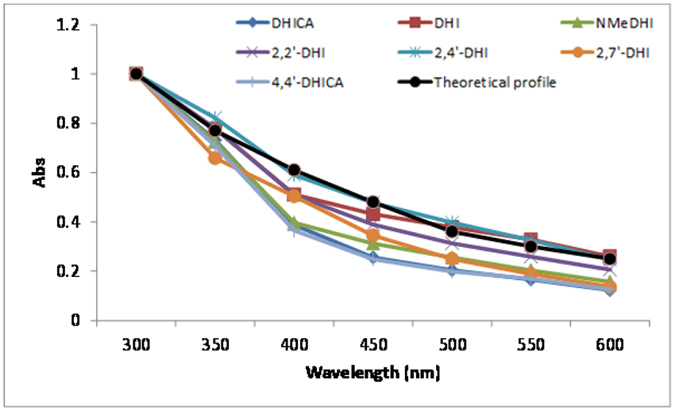
Relative absorbance of the oxidation mixtures of the indole compounds at 24 h over the UV-visible region in the presence of 1% PVA (values normalized against the absorbance at 300 nm for each mixture).

To compare the shapes of the final melanin curves in Fig. 5 with those of the monomers, absorbance data at fixed visible wavelengths were normalized against the UV band at 300 nm and the resulting traces were matched against the theoretical monotonic profile (Fig. 6).

Data showed a strongly similar trend for DHICA and its 4,4′-dimer, but markedly different graphs in the case of DHI and its dimers. Noticeable differences were likewise observed in the spectra from the three dimers, with visible chromophore intensity decreasing in the order 2,4′- >2,2′- >2,7′-isomers. Comparison of data in the presence and in the absence of PVA indicated marked variations in the curve of DHI melanin, consistent with a dominant scattering effect, but not in the case of DHICA melanin (see also Supplementary Fig. 13).

Since increased chemical disorder with respect to reference compounds, e.g. 5,6-dihydroxyindoles, was anticipated as a consequence of the oxidation of precursors that lie upstream in the eumelanin pathway, the overall picture of eumelanin formation was completed by separate experiments run on 3,4-dihydroxyphenylalanine (DOPA) and dopamine (DA) as the early catecholamine precursors. Since DHI and DHICA are produced from catecholamines by a 4-electron oxidation process, in these experiments the oxidant stoichiometry was adjusted to keep conversion to 5,6-dihydroxyindoles into account. As expected (Supplementary Figs 14 and 15) this analysis revealed marked differences in the early stages of DOPA and DA oxidation compared to DHI or DHICA, including chiefly the lack of detectable melanochrome formation.

The most significant difference between DOPA and DA melanin (polydopamine) was the lower visible absorption observed in the latter case. This can be explained in view of the lower tendency of DA to cyclize to indole units compared to DOPA.

From these absorbance data, formal extinction coefficients (ε_*f*_) were determined at 450, 550 and 650 nm for the final eumelanin chromophores produced from the monomeric compounds in the presence of PVA, i.e. under conditions of limited precipitation and scattering (Table 1). Formal extinction coefficients were defined as:


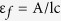


where A is the absorbance, c is the initial monomer concentration and l is the optical path in cm.

Several important observations and trends were revealed from Table 1:

- DHI melanin (24 h) exhibits formal extinction coefficients that are two times higher than those of DHICA melanin at 650 nm.
- 650/550 nm coefficient ratios for DHICA are invariably lower than for DHI throughout oxidation;
- 550/450 nm and 650/550 nm ratios at 5 min, taken as relative indices of melanochrome intensity, show respectively higher and lower values in the case of DHICA compared to DHI, consistent with a more defined band shape;
- The decrease in the 550/450 nm coefficient ratio and the increase in the 650/550 nm coefficient ratio on passing from 5 min to 2 h, taken as an index of band broadening under limited influence of scattering, are both much more consistent in the case of DHICA.

Overall, these results confirmed the inhibitory effect of the carboxyl group in DHICA on all phases of visible chromophore development relative to DHI, i.e. at the melanochrome phase (lower extinction coefficient at 5 min at 550 nm than DHI), at the band-broadening phase and on scattering. It is also apparent that DHICA oxidation leads to a more defined melanochrome chromophore, which suffers more evident broadening and flattening with time compared to DHI.

### The role of redox disorder

Redox disorder was herein related to the possible occurrence of each structural component (identified by the σ-scaffold) at different oxidation levels. For example, dimers can in principle exist in the entire gradational range of redox states comprised between the fully reduced (4 H) species and the oxidized fully quinonoid species (0 H). It can be easily deduced that, apart from isomerism, each oligomer can give rise to n + 1 redox species where n is the number of OH groups. Although it was difficult to probe redox disorder compliant to the above definition, indirect information about the average redox state was sought through experiments aimed at probing susceptibility of eumelanin final chromophore to modification by oxidants, reducing agents and monomers.

Exposure to chemical oxidants such as sodium periodate or potassium ferricyanide of final eumelanin samples produced in the presence of PVA did not cause appreciable effects on visible chromophores. Persistence of the ferricyanide maximum after the addition ruled out moreover the occurrence of oxidation processes unrelated to chromophoric species. Conversely, addition of sodium dithionite or sodium borohydride to eumelanin samples produced in the presence of PVA caused a detectable decrease in the visible absorption of all eumelanin samples examined associated to an increase in the UV band, this latter more marked in the case of DHI (Supplementary Fig. 16). These data supported the view that reducible quinonoid species are important determinants of the visible chromophores, despite the failure to attain complete color suppression. In relation to this latter observation, it is possible that the wrapping of eumelanin components, within PVA chain coils, hinders attack of reductants. Separate experiments carried out on oxidation mixtures produced in the absence of PVA and using an excess of either of the reducing agents showed comparable decrease of the absorption in the visible region (Supplementary Fig. 16). It is relevant to notice that acidification to pH 2 caused a detectable ipsochromic shift of the visible chromophore of eumelanins with a concomitant bathochromic shift of the UV band (Supplementary Fig. 17), while no effect was measured upon rising the pH to 9. These data suggested that chromophoric species are present in an ionized quinonoid form at neutral pH.

Although no direct evidence could be obtained for the presence of reduced catechol units susceptible to oxidation, these experiments overall confirmed the fundamental contribution of oxidized quinonoid units to eumelanin chromophore.

To further probe eumelanin redox properties, in a final set of experiments eumelanin samples prepared by ferricyanide oxidation of DHI or DHICA were exposed to the same concentration of the relevant monomer. In the case of DHICA melanin, virtually additive spectra could be recorded after the addition (see Supplementary Fig. 18) without significant monomer conversion or visible chromophore change in air or under an argon atmosphere either in the presence or in the absence of PVA. Analysis of the spectra obtained in the case of DHI showed likewise no appreciable changes on addition of the monomer provided that the additive contribution of the separate chromophores is duly considered (see Supplementary Fig. 18). These data would suggest no apparent redox activity for either melanins.

### Computational modeling of intermolecular interactions

The overall set of spectrophotometric experiments reported so far strongly pointed to intermolecular interactions between oxidatively-generated visible species as the main mechanism underlying the slow line-broadening processes that gradually override the intrinsic absorption features of melanochromes. The weaker extinction coefficients of DHICA melanin compared to DHI melanin were consistent with the generation of poorly-delocalized intrinsic chromophores less amenable to strong intermolecular interactions and redox re-equilibration.

To confirm these conclusions, a series of computational studies were carried out with a view to probing the generation of extrinsic visible chromophores via intermolecular interaction of dimers from DHI and DHICA at different oxidation levels.

Preliminary calculations (Supplementary Figs 19–24) confirmed that DHI and DHICA, in either monomeric or dimeric form do not show any appreciable absorption in the visible region in the reduced catechol form (**HQ)**, whereas the 2-electron oxidation products of DHI and DHICA dimers (**Q**) exhibit markedly different absorption spectra. In agreement with experimental findings^14^,^21^, oxidation forces DHI-dimers into perfectly planar structures with strong visible absorption properties, whereas the DHICA dimer fails to adopt a planar conformation and, hence, to strongly absorb in the visible range. This simple observation however does not provide an entirely satisfactory explanation to the broadband visible absorption exhibited by DHI melanins, and to the significant rise of visible absorption in DHICA melanins from monomer and dimer (Figs 3, 4 and 5). On this basis, a more robust rationalization of the absorption process required consideration of extra factors in addition to the intrinsic absorption properties of dimer intermediates. Both the influence of PVA on eumelanin chromophore development and recent computational studies^17^,^22^, revealing the appearance of a broad absorption band from **HQ**/**Q**, or a similar pair, in a closely stacked configuration, suggested a possible role of non-covalent intermolecular interactions between the intrinsic chromophores.

To further support this hypothesis, a number of stacked configuration were assembled, for each of the investigated dimers (Supplementary Figs 22 and 25), by approaching to each other the reduced (**HQ**) and 2-electron oxidation forms (**Q**). Selection of these models was supported by previous evidence on the coexistence of oxidized and reduced species in eumelanin chromophore^10^,^21^.

**Figure 7:**
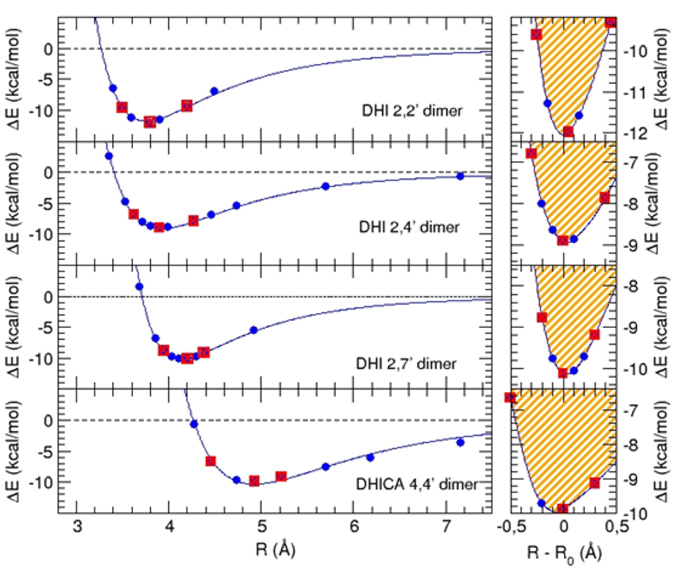
Left panels: interaction energy curves computed at MP2 level for stacked DHI and DHICA dimer pairs. Right panel: Allowed displacements along the stacking direction at room temperature. The conformers (displayed in Supplementary Fig. 25) selected for spectrum calculation are evidenced with red boxes.

The reliability and accessibility of such conformations was considered by accurately computing their interaction energy as a function of the separation of the **HQ** and **Q** molecular planes (left panels of Fig. 7). Though all conformers proved rather stable (~10 kcal/mol), MP2 calculations of **HQ**/**Q** complexes predicted an important difference between DHI and DHICA dimers: while the former attained stability around 4 Å, the latter were stable at significantly larger distance. Due to the high computational cost, no geometry optimization was performed at this level on the **HQ**/**Q** dimer pairs (with the sole exception of the 2,4′-dimer as test case, see below).

Three stacked conformers for each investigated species (red boxes in Fig. 7) were then selected and their absorption spectra computed at TD-DFT level.

**Figure 8:**
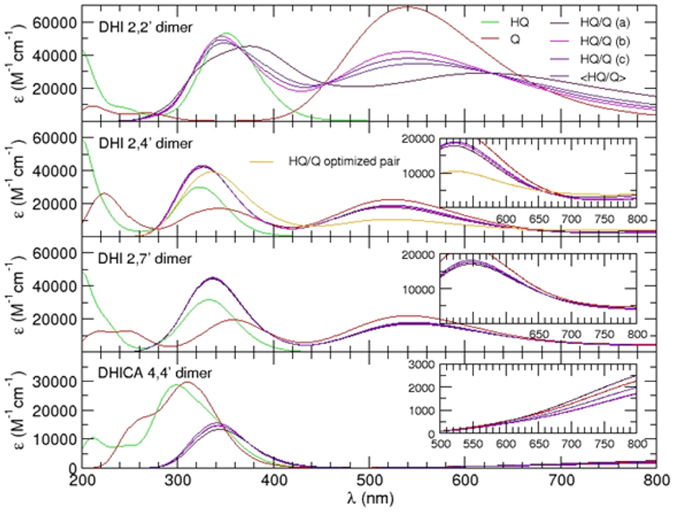
Computed absorption spectra for the HQ (green), Q (red) and HQ/Q (indigo, averaged over three conformers) species of the investigated dimers. The absorption of each HQ/Q stacked conformer at short (**a**), minimum (**b**) and large (**c**) distance, is reported separately. In the insets, the visible region between 500 and 800 nm is highlighted.

Scrutiny of panels in Fig. 8 showed that all **Q** forms of DHI dimers exhibit intense visible bands, which were affected by stacking in the **HQ**/**Q** complexes. The influence of stacking was more evident in the planar 2,2′-dimer stack and involved a red-shift of main bands accompanied, especially at short stacking distances, by broadening and increased low-energy absorption. Although the effects were less discernible in the **HQ**/**Q** complexes from the 2,4′- and 2,7′-dimers, a supplementary geometry optimization performed on the 2,4′-dimer indicated that the **HQ**/**Q** pair can access to more favourable stacking energies (~15 kcal/mol) and consequently, to smaller intermolecular distances, causing a distinct effect of band broadening. This finding suggested that allowing for geometry optimization might lead to more significant enhancements than reported in this preliminary survey. The most efficient dimer stacking interactions predicted for the 2,2′-isomer was compatible with the evolution of chromophores observed in Supplementary Fig. 9 in the absence of PVA.

On the other hand, a remarkable effect due to stacking was predicted for the DHICA dimer. In that case, the oxidized **Q** form gave a chromophore virtually superimposable to that of **HQ**. However, as the dimers approached to form the **HQ**/**Q** complex, a marked bathochromic shift occurred in the UV band, bearing no resemblance to the behavior of DHI dimers, accompanied by the appearance of a very flat band at the low energy end of the visible spectrum. This band, which was clearly missing in the spectra of separated **HQ** and **Q** forms, provided support to the proposed development in DHICA melanin of stabilizing intermolecular interactions causing the appearance of low energy transitions in the visible range.

## Discussion

The integrated experimental and computational bottom-up approach reported herein was aimed at obtaining a chemical background in which to frame currently accepted theories about eumelanin chromophore buildup and the origin of broadband absorption spectrum. The main findings of this study can be summarized as follows:

1. Eumelanin chromophore development from DHI involves complex dynamics which reflects the fast generation of visible chromophores (“melanochromes”, phase I), up to a point beyond which further addition of oxidants has no detectable effect. A much slower band broadening process follows (phase II), which does not depend on oxygen or oxidants and results eventually in the deposition of a precipitate (phase III).
2. DHICA melanin shows much less intense visible absorption compared to DHI melanin, even in the presence of PVA, confirming the influence of the 2-carboxyl group^23^. Spectrophotometric oxidation courses virtually superimposable to those of DHICA were observed from the 4,4′-dimer of DHICA.
3. Melanochrome chromophores from DHI and isomeric dimers of DHI displayed marked differences, which tended to even out only in part in the final eumelanin spectra.
4. Both melanochromes and eumelanin final chromophores in PVA are not affected by alkali but undergo blue-shifts in acids. They can be reduced in part with sodium dithionite but are not affected by oxidants, and show significant deviations from the theoretical monotonic profile.
5. QM calculations mimicking the stacking processes between **HQ**/**Q** dimer pairs revealed formation of stable complex pairs, able to generate large bands in the visible region depending on the efficiency of stacking. This latter process was disfavored in the DHICA dimer, because its twisted scaffold prevents the close stacking distances found for DHI dimers. Average intermolecular stacking forces were estimated in the order of 10 kcal/mol or stronger.

Overall, these observations contribute to compose, to the best of our knowledge, the most complete experimental and theoretical background of comparative studies on eumelanin chromophore so far available.

**Figure 9:**
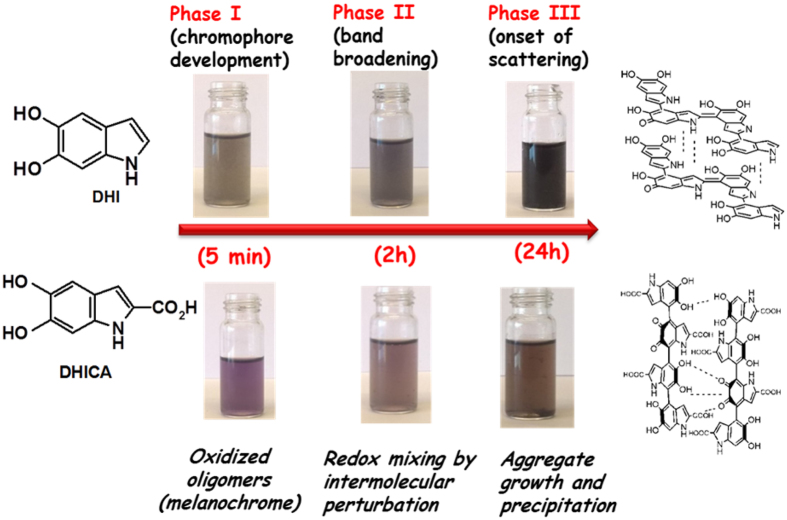
Overview of the color development and the underlying processes associated to the three phases of the oxidation of 5,6-dihydroxyindole eumelanin precursors.

In this picture, rapid melanochrome formation (phase I) can be attributed to the intrinsic chromophores of oligomer ensembles in a stable oxidation state. The subsequent oxidant-independent band broadening process (phase II) would reflect an intermolecular reorganization/equilibration of redox state driven by optimization and slow rearrangement of preliminary stacking interactions during oligomer growth. Resistance to oxidation and in part to reduction of these chromophores would be a consequence of extensive redox mixing toward extensive three-dimensional electronic delocalization. Stabilization of all individual chromophores into aggregates would cause loss of the early intrinsic optical features of melanochromes through mutual perturbations and their merging into broadband absorptions, which hence would be largely extrinsic in nature. The intermolecular nature of these effects would be indirectly supported by the inhibitory effect of PVA on melanochrome decay during DHICA melanin synthesis. Finally, development of black color is largely associated to attainment of the tightest aggregation possible, i.e. the solid state, and in consequence of the strongest π-electron stabilization in the solid state (phase III) (Fig. 9). It is tempting to argue from data in Fig. 5 that differences in the shape of melanin final curves are largely due to scaffold-controlled differences in the *intrinsic* absorption properties (compare e.g. spectra of DHI versus its dimers), whereas *extrinsically* determined contributions based on intermolecular interactions tend to even out differences (see the spectra of DHICA melanin versus its 4,4′-dimer).

Evidence in support of the proposed redox equilibration via slow intermolecular interaction of eumelanin components is provided by a recent study in which an organic electrochemical transistor (OECT) was used to investigate the electrical properties of eumelanin biopolymers^24^. Gate current measurements on fine aqueous suspensions of DHI eumelanin revealed a well detectable hysteretic response which gradually decreased over 5 cycles. These data were consistent with evolution of DHI melanin from a far-from-the-equilibrium redox state toward a more stable electronic arrangement promoted by redox exchange with the gate electrode.

Overall, the results of the present study and previous data concur to indicate that melanochrome components displaying oligomeric scaffolds at mixed oxidation states, i.e. containing both catechol and quinone moieties or related extended quinone moieties, provide the most reasonable candidate structures for intermolecular interactions underpinning excitonic effects in the proposed geometric order and disorder model.

In the free ionized form, the carboxyl group in DHICA decreases but not prevents the generation of melanochrome, which is consistently less intense relative to DHI melanin. This difference would reflect the inability of DHICA oligomers to access to planar conformations, thus decreasing intramolecular π-electron delocalization and intermolecular chromophore perturbation by stacking interactions. It can be concluded that the final visible chromophores would reflect *both intrinsic and extrinsic* contributions in DHI melanin, as enhanced by largely planar conformations, *but mainly extrinsically-defined* contributions in DHICA melanin due to weak intermolecular interactions at catechol-quinone contact sites.

Besides contributing to delineate the main chemical processes underlying the dynamics of eumelanin chromophore buildup, the results reported herein would definitively settle some key issues and false beliefs that have conditioned progress toward elucidation of eumelanin optical properties.

In the first place, it is clearly demonstrated that eumelanin broadband chromophore is not universally identified by the monotonic and featureless decay profiles commonly reported in the literature, but shows appreciable deviations due to specific absorption features controlled by monomer structure.

In the second place, the efficiency of intermolecular interactions leading to redox equilibration and stacking is an important determinant of chromophore broadening in an oxidation independent manner. A major implication is that extrinsic absorption contributions caused by intermolecular stacking interactions may partly override the intrinsically-defined contributions related to structural complexity set at the melanochrome stage.

Although the relevance of these results to eumelanin optical properties *in vivo* within the melanosomes remains to be assessed, the new background of structure-property relationships emerging from this study may guide the rational design of eumelanin-inspired functional materials tailored to specific biomedical, dermocosmetic or technological applications^3^.

## Methods

### Materials

3,4-Dihydroxy-l-phenylalanine (l-dopa), dopamine hydrochloride, hydrogen peroxide (30% vol⁄vol), potassium ferricyanide, sodium periodate, nickel sulfate eptahydrate, copper acetate, ceric ammonium nitrate (CAN), sodium dithionite, sodium borohydride, horseradish peroxidase (HRP; donor: H_2_O_2_ oxidoreductase, EC 1.11.1.7) and sodium *tert*-butoxide were purchased from Sigma-Aldrich. All solvents were HPLC grade. Bidistilled deionized water was used throughout the study.

### Analytical methods

HPLC analyses were performed on an Agilent 1100 instrument equipped with a UV detector set at 254 nm. An octadecylsilane-coated column, 250 mm × 4.6 mm, 5 μm particle size (Phenomenex Sphereclone ODS) at 0.7 mL/min was used with the following gradient: 1.5% formic acid (eluant a)/methanol (eluant b) from 5 to 90% b, 0–45 min. Preparative HPLC was carried out on an instrument coupled with a UV detector set at 280 nm using an Econosil C18 (10 micron, 22 × 250 mm), at 15 mL/min, and 1.5% formic acid: methanol 85:15 as the eluant. UV-vis spectra were recorded on a Jasco V-730 Spectrophotometer.

### Preparation of 5,6-dihydroxyindole monomers

5,6-dihydroxyindole (DHI)^25^, 5,6-dihydroxyindole-2-carboxylic acid (DHICA)^25^ and 5,6-dihydroxy-*N*-methylindole (N-MeDHI)^26^ were prepared as described.

### Preparation of 5,6-dihydroxyindole dimers

2,2′-biindolyl^27^, 2,4′-biindolyl and 2,7′-biindolyl^28^ from DHI were prepared as previously described with slight modifications. Briefly, a solution of DHI (300 mg, 2.0 mmol) in 0.05 M phosphate buffer (pH 6.8) (120 mL) was treated with horseradish peroxidase (36 U/mL) and H_2_O_2_ (266 μL of a 30% solution, 2.3 mmol). After 25 s, the oxidation reaction was stopped by addition of a solution of sodium dithionite and rapidly extracted with ethyl acetate. After acetylation of the residue obtained following evaporation of the combined organic layers with acetic anhydride-pyridine overnight at room temperature the mixture was fractionated by column chromatography (gradient elution, CHCl_3_−ethyl acetate from 9:1 to 6:4) to afford the 2,7′-biindolyl (45 mg, 10% yield, >95% pure) and the 2,4′-biindolyl (53 mg, 11%, >95% pure) as acetylated derivative. For isolation of the 2,2′-biindolyl as the *O*-acetyl derivative (30 mg, 12% yield) DHI (150 mg, 1.0 mmol) was oxidized in air in 0.05 M HEPES buffer (pH 7.5) in the presence of NiSO_4_·7H_2_O (560 mg, 2.0 mmol). After acetylation treatment of the ethyl acetate extracts the residue was taken up in acetone and the product was recovered by filtration as white prisms. 4,4′-Biindolyl from DHICA was prepared as previously described^29^ with modifications. A solution of DHICA (100 mg, 0.51 mmol) in 0.1 M HEPES buffer (pH 7.5) (495 mL) was treated with copper acetate (1 molar equivalent) and the reaction mixture was taken under vigorous stirring. After 10 min, the oxidation reaction was stopped by addition of sodium dithionite, acidified to pH 2 and rapidly extracted with ethyl acetate. The residue obtained following evaporation of the combined organic layers was fractionated by preparative HPLC (16 mg, 16% yield, >90% pure).

### Oxidation of 5,6-dihydroxyindole monomers and dimers

15 μL of a 10 mM solution of the appropriate indole monomer or 4,4′-DHICA-dimer were added to 3 mL of 0.1 M phosphate buffer (pH 7.0) to reach a final concentration of 50 μM followed by 2 molar equivalents of potassium ferricyanide (30 μL of a 10 mM solution in water). In the case of DOPA and DA the reaction was carried out with 6 molar equivalent of ferricyanide. The reaction mixtures were taken under vigorous stirring and periodically analyzed by UV-vis spectrophotometry. Spectra were registered at 5 min, 2 h and 24 h. After 5 min in air, reaction mixtures were degassed and kept under stirring in an argon atmosphere.

In other experiments the reaction was performed:

- In the presence of sodium periodate (1 molar eq.).
- In the presence of CAN (2 molar eq.) in 0.1 M phosphate buffer (pH 3.0).
- Adding sodium dithionite or sodium borohydride after the addition of potassium ferricyanide (2 or 40 molar equivalents).

When required, oxidation mixtures were filtered through a 0.45 micron nylon membrane.

Substrate consumption was determined by HPLC analyses under the conditions described above.

When required the reaction was carried in 0.1 M phosphate buffer (pH 7.0) containing 1% poly(vinyl alcohol) (PVA) to prevent polymer precipitation.

In the case of acetylated dimers the following protocol was followed: 5 mL of a 3.6 mM solution of the acetylated dimer in methanol were treated under an argon atmosphere with sodium *tert*-butoxide (8 molar equivalents) and after 5 min the pH of the solution was taken to 2 by addition of 4 M HCl. 42 μL of the resulting mixture was added to 3 mL of 0.1 M phosphate buffer (pH 7.0) to reach a final concentration of 50 μM followed by 2 molar equivalents of potassium ferricyanide.

### Computational studies

For both hydroxy (**HQ**) and “quinone” (**Q**) forms of all considered species (2,2′, 2,4′, 2,7′-DHI and 4,4′-DHICA dimers), a complete geometry optimization was carried out *in vacuo* at DFT level, employing the CAM-B3LYP functional with 6–311 + G(d,p) level.

The interaction energy in all **HQ**/**Q** stacked pairs was computed at MP2 level, using a specifically modified 6–31G* basis set, where the polarization exponents on heavy atoms were optimized against CCSD(T)@cbs reference data for similar molecules^17^,^22^. The employed exponents are 0.25, 0.44 and 0.37 for C, O and N atoms, respectively. The method has been shown to give very accurate results for the interaction energy of both quinhydrone^22^ and DHI related pairs^17^.

The geometry optimizations of the 2,4′ **HQ**/**Q** complex was carried out through the in house software Poldo^30^, which exploits the Fragmentation Reconstruction Method (FRM)^30^,^31^ to compute the interaction energy. The latter was again computed at MP2/6–31G(0.25,0.44,0.37) level.

For all investigated compounds, vertical transition energies were computed at TD-DFT level, using the B3LYP functional with the Dunning’s cc-pvDz basis set. The spectra were obtained by convoluting the transition energies calculated for the first 25 excited stated with Gaussian functions with 0.33 eV HWHM. In all TD calculations, the solvent effect was accounted for through the polarizable continuum model (PCM).

The absorption computed at low wavelength (<250 nm) is much lower than in the experiment due to the limited number of excited eigenstates considered. Test calculations performed for the HQ/Q 2,2′-dimer pair (see Supplementary Fig. 26) show that the absorption in the region increases consistently with the number of excited states considered in the calculations.

## Additional Information

**How to cite this article:** Micillo, R. *et al*. Eumelanin broadband absorption develops from aggregation-modulated chromophore interactions under structural and redox control. *Sci. Rep.* **7**, 41532; doi: 10.1038/srep41532 (2017).

**Publisher's note:** Springer Nature remains neutral with regard to jurisdictional claims in published maps and institutional affiliations.

## Supplementary Material

Supplementary Material

## Figures and Tables

**Figure 1:**
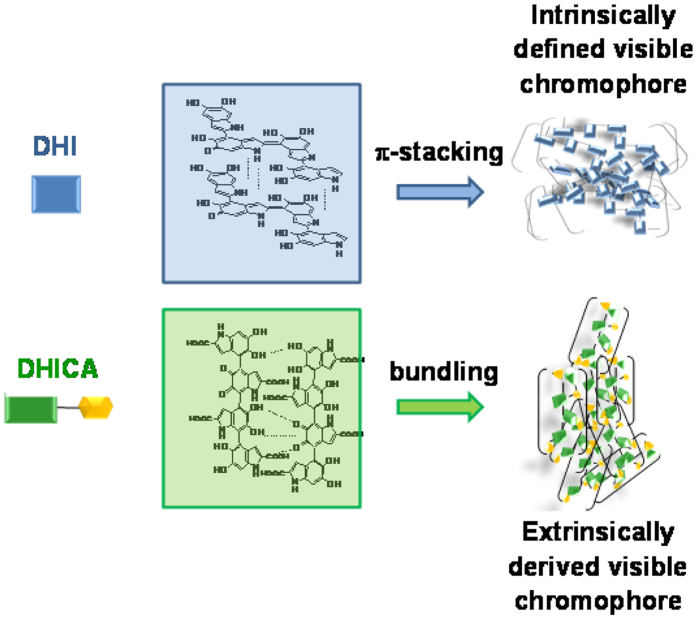
Disentangling eumelanin absorption properties: intrinsic and extrinsic contributions.

**Figure 2:**
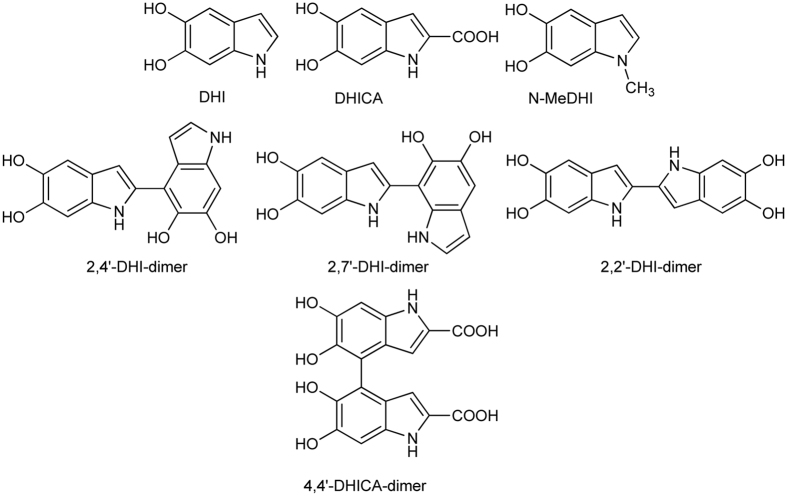
Structures of eumelanin precursors investigated in this study.

**Table 1:** Formal extinction coefficients at different wavelengths and reaction times of the eumelanin cromophores investigated.

Precursor (50 μM)	ε_*f*_ (450)	ε_*f*_ (550)	ε_*f*_ (650)	ε_*f*_ ratio 550/450	ε_*f*_ ratio 650/550
5 min					
DHI	2460	2909	2343	1.18	0.81
DHICA	1788	2618	1176	1.46	0.45
DOPA	3059	1267	256	0.41	0.20
DA	2589	1419	440	0.55	0.17
2 h					
DHI	3210	3369	2787	1.05	0.83
DHICA	1957	1990	1212	1.02	0.61
DOPA	3106	2478	1956	1.62	0.79
DA	1529	661	306	0.43	0.20
24 h					
DHI	3423	2603	1467	0.76	0.56
DHICA	2304	1493	705	0.65	0.47
DOPA	4042	2441	1316	0,60	0.54
DA	1915	967	344	0.50	0.36
