# Supplementary material for: Eumelanin broadband absorption develops from aggregation-modulated chromophore interactions under structural and redox control
Source: Sci Rep. 2017 Feb 2;7:41532. doi: 10.1038/srep41532 (PMC5288692; doi:10.1038/srep41532)
Supplement: Supplementary Material [file srep41532-s1.pdf]

Eumelanin broadband absorption develops from aggregation-modulated chromophore interactions under structural and redox control

Raffaella Micillo[1], Lucia Panzella[2], Mariagrazia Iacomino[2], Giacomo Prampolini[3], Ivo Cacelli[3,4], Alessandro Ferretti[3], Orlando Crescenzi[2], Kenzo Koike[5], Alessandra Napolitano[2], Marco d'Ischia[2,*]

[1] Department of Clinical Medicine and Surgery, University of Naples Federico II, I-80131 Naples, Italy.

[2] Department of Chemical Sciences, University of Naples Federico II, I-80126 Naples, Italy.

[3] Istituto di Chimica dei Composti Organometallici (ICCOM-CNR) , Area della Ricerca, I-56124 Pisa, Italy.

[4] Dipartimento di Chimica e Chimica Industriale, Università di Pisa, I-56124 Pisa, Italy.

[5] Hair care Products Research Laboratories, Kao Corporation, Tokyo 131-8501, Japan.

Correspondence and requests for materials should be addressed to MdI (dischia@unina.it)

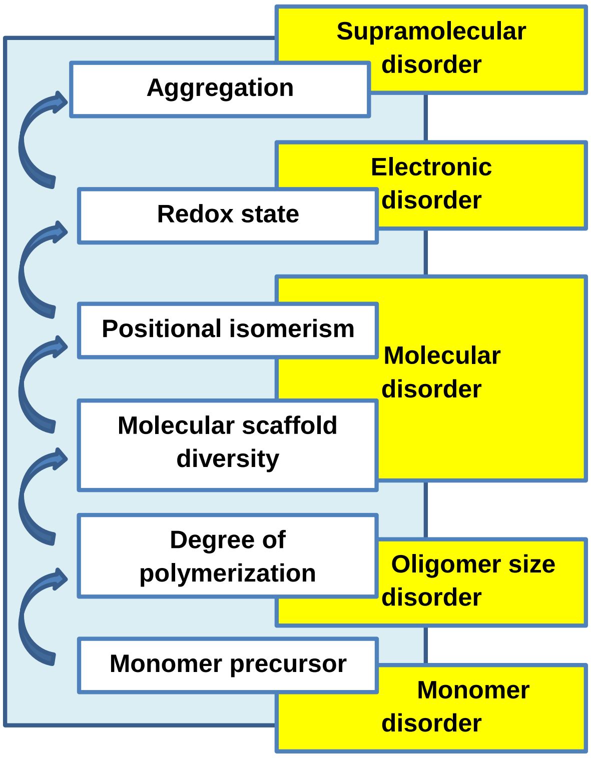

Supplementary Figure 1| Main levels of disorder in synthetic eumelanins.

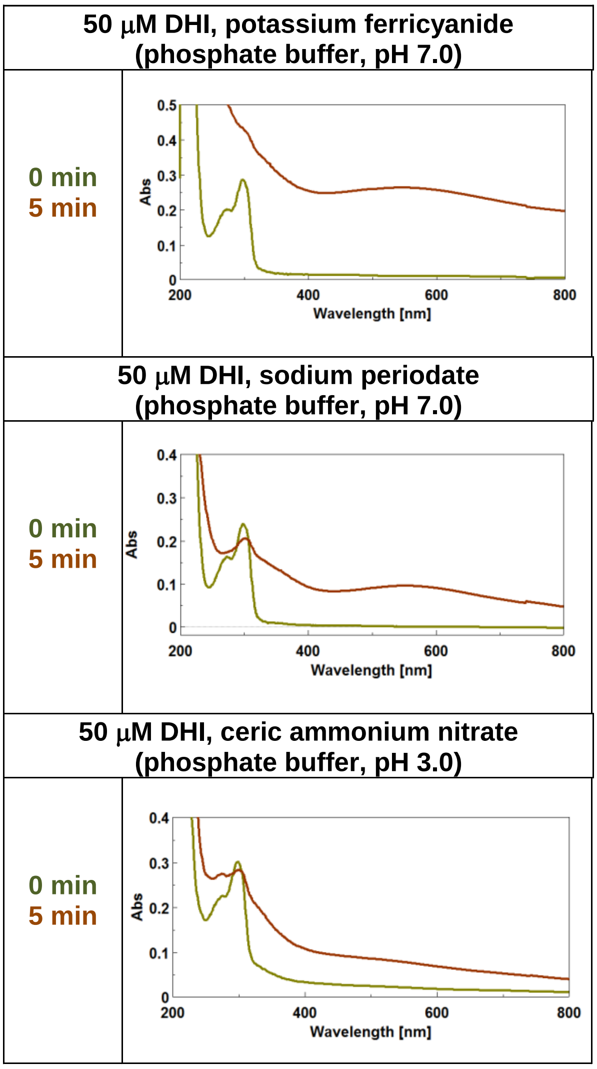

Supplementary Figure 2| Generation of chromophores from DHI using different oxidizing systems, namely potassium ferricyanide or sodium periodate, in phosphate buffer at pH 7.0, ceric ammonium nitrate (CAN) in phosphate buffer at pH 3.0.

50 µM DHI	Phosphate buffer pH 7.0<br>without PVA	Phosphate buffer pH 7.0<br>1% PVA
0 min<br>5 min<br>2 h<br>24 h	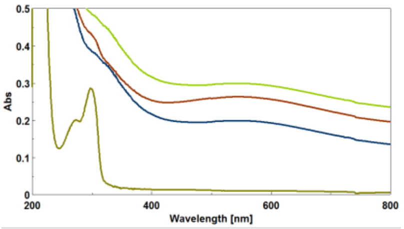	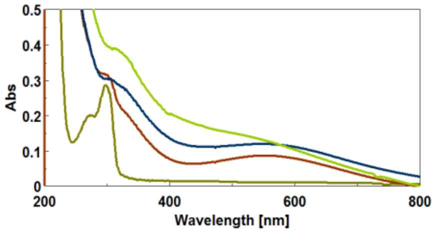
5 min<br>No filtr.<br>Filtr.	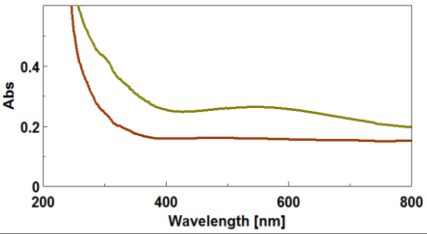	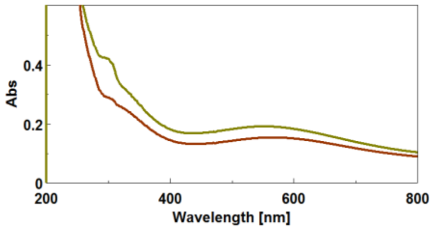
2h<br>No filtr.<br>Filtr	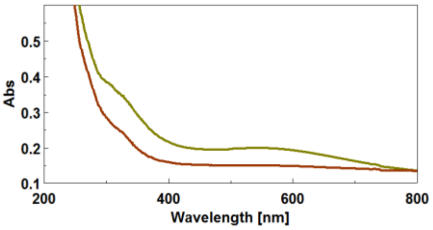	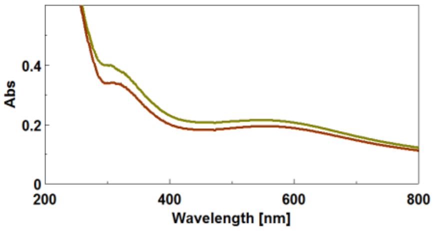
24 h<br>No filtr.<br>Filtr.	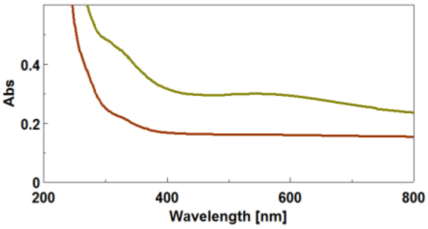	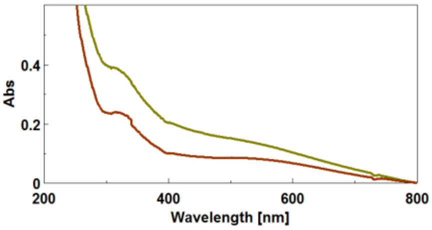

Supplementary Figure 3| Generation and evolution of chromophores from DHI oxidation by ferricyanide (2 molar equivalents) in the presence and in the absence of 1% PVA, with or without filtration.

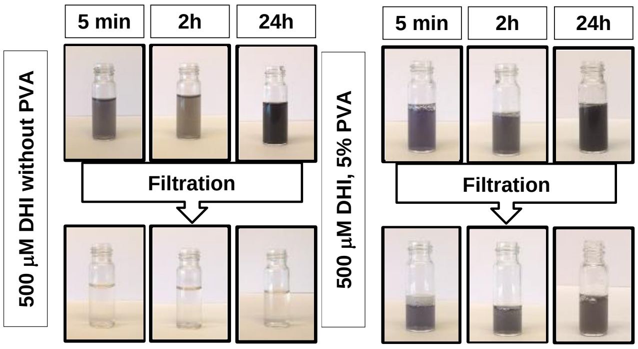

Supplementary Figure 4| Digital pictures of the oxidation mixtures obtained by ferricyanide oxidation of DHI over 24 h in the absence and in the presence of 5% PVA.

50 µM N-MeDHI	Phosphate buffer pH 7.0 without PVA	Phosphate buffer pH 7.0 1% PVA
0 min<br>5 min<br>2 h<br>24 h	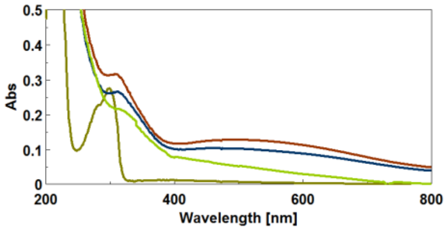	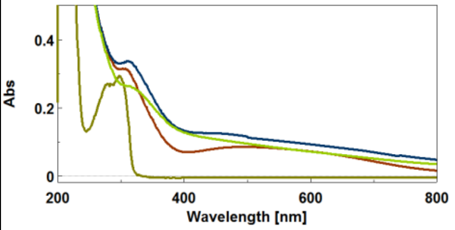
5 min<br>No filtr.<br>Filtr.	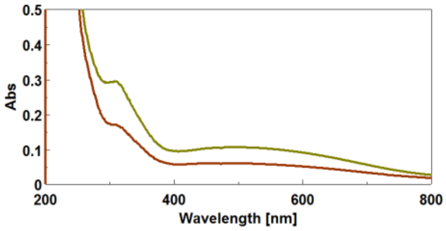	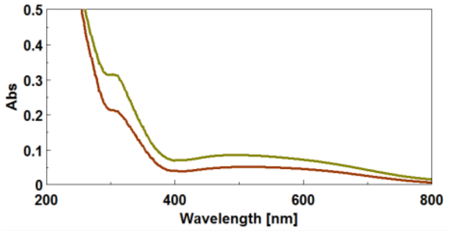
2h<br>No filtr.<br>Filtr	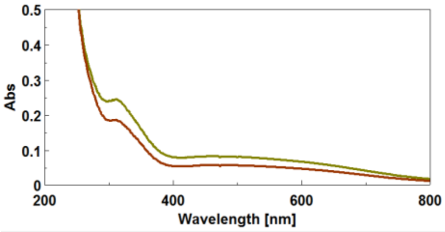	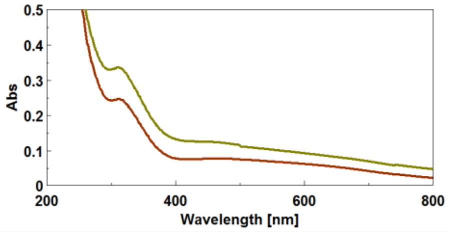
24 h<br>No filtr.<br>Filtr.	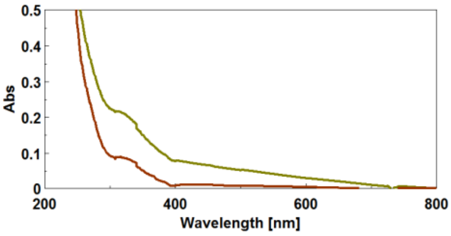	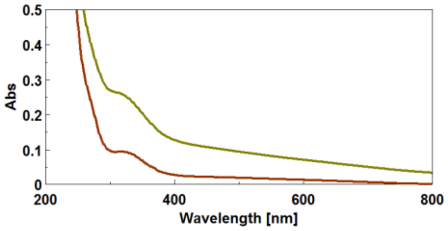

Supplementary Figure 5| Generation and evolution of chromophores from oxidation of N-MeDHI by ferricyanide in the absence and in the presence of 1% PVA, with or without filtration.

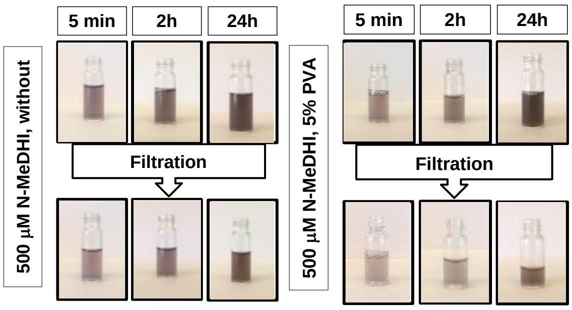

Supplementary Figure 6| Digital pictures of mixtures obtained by ferricyanide oxidation of N-MeDHI over 24 h in the absence and in the presence of 5% PVA.

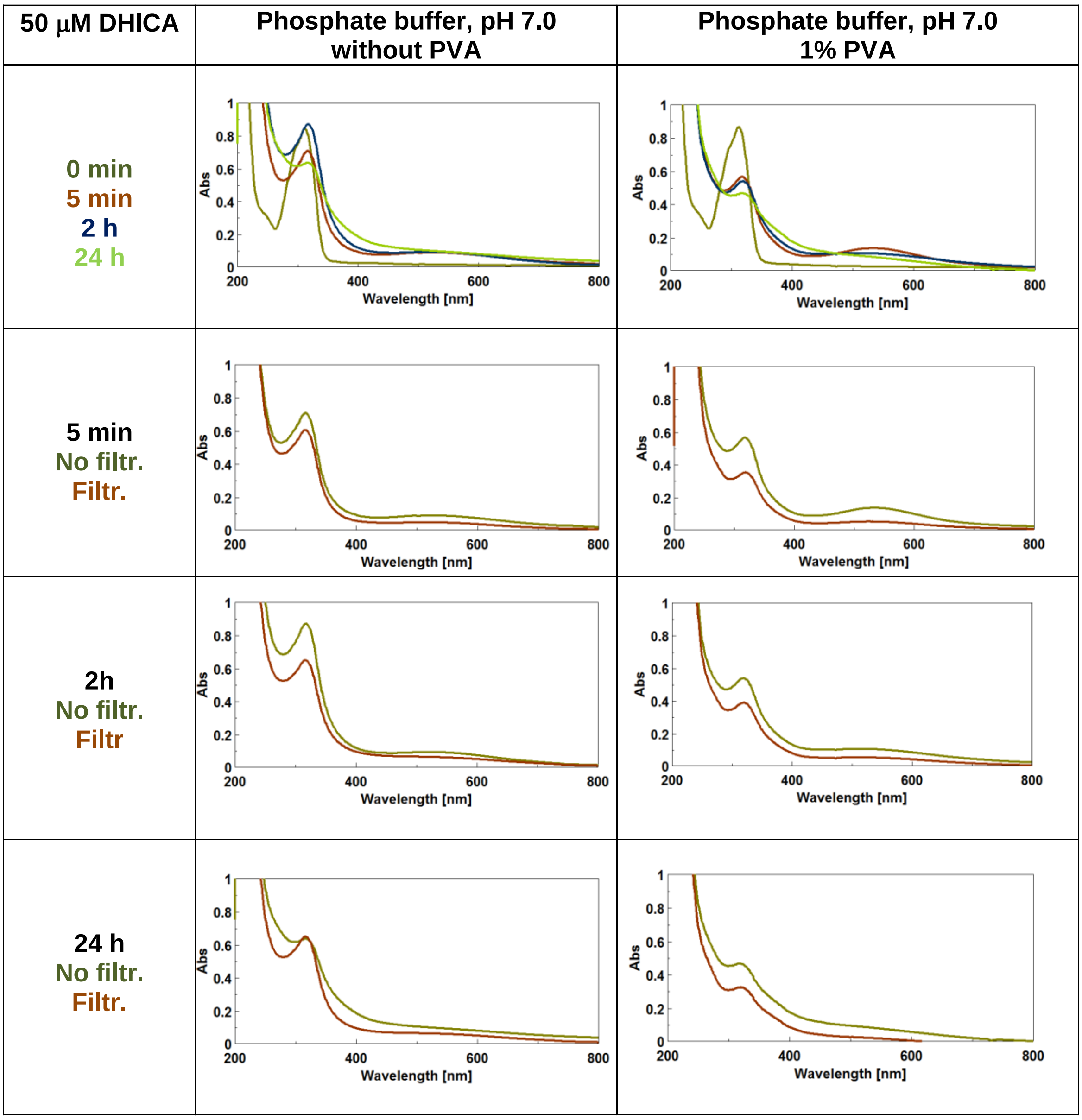

Supplementary Figure 7| Generation and evolution of chromophores from oxidation of DHICA by ferricyanide in the absence and in the presence of 1% PVA, with or without filtration.

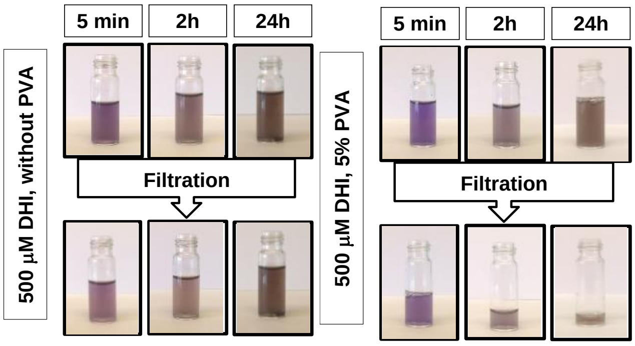

Supplementary Figure 8| Digital pictures of mixtures obtained by ferricyanide oxidation DHICA over 24 h in the absence and in the presence of 5% PVA.

50 µM 2,2’-DHI	Phosphate buffer, pH 7.0 without PVA	Phosphate buffer, pH 7.0 1% PVA
0 min<br>5 min<br>2 h<br>24 h	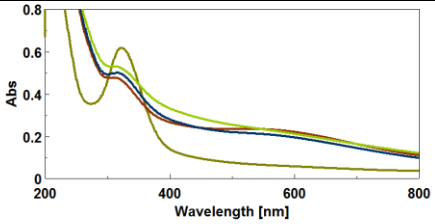	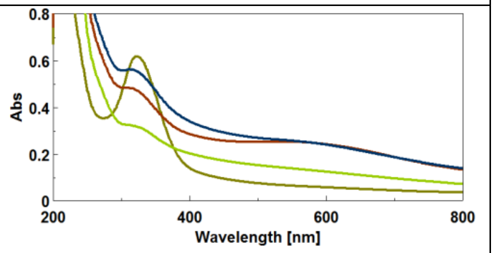
5 min<br>No filtr.<br>Filtr.	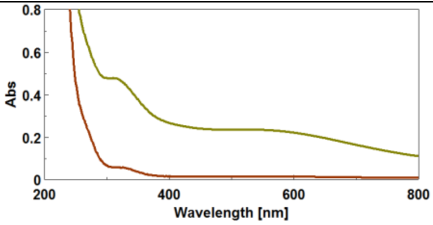	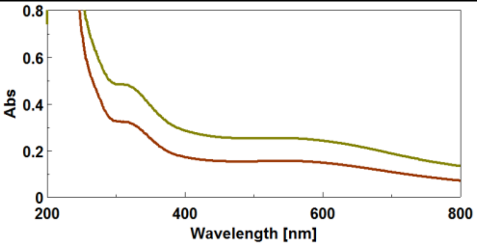
2 h<br>No filtr.<br>Filtr	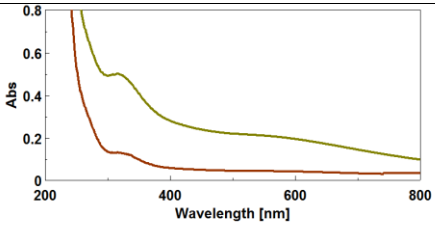	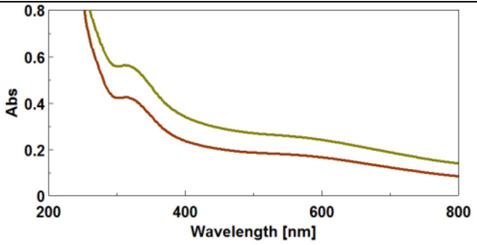
24 h<br>No filtr.<br>Filtr.	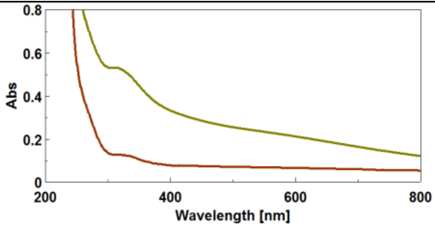	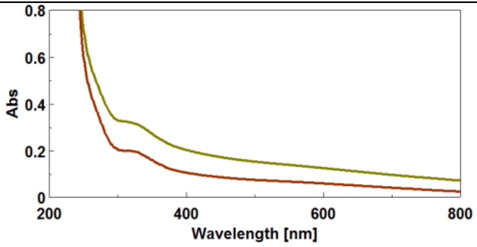

Supplementary Figure 9| Generation and evolution of chromophores from oxidation of 2,2’-DHI dimer by ferricyanide in the absence and in the presence of 1% PVA, with or without filtration.

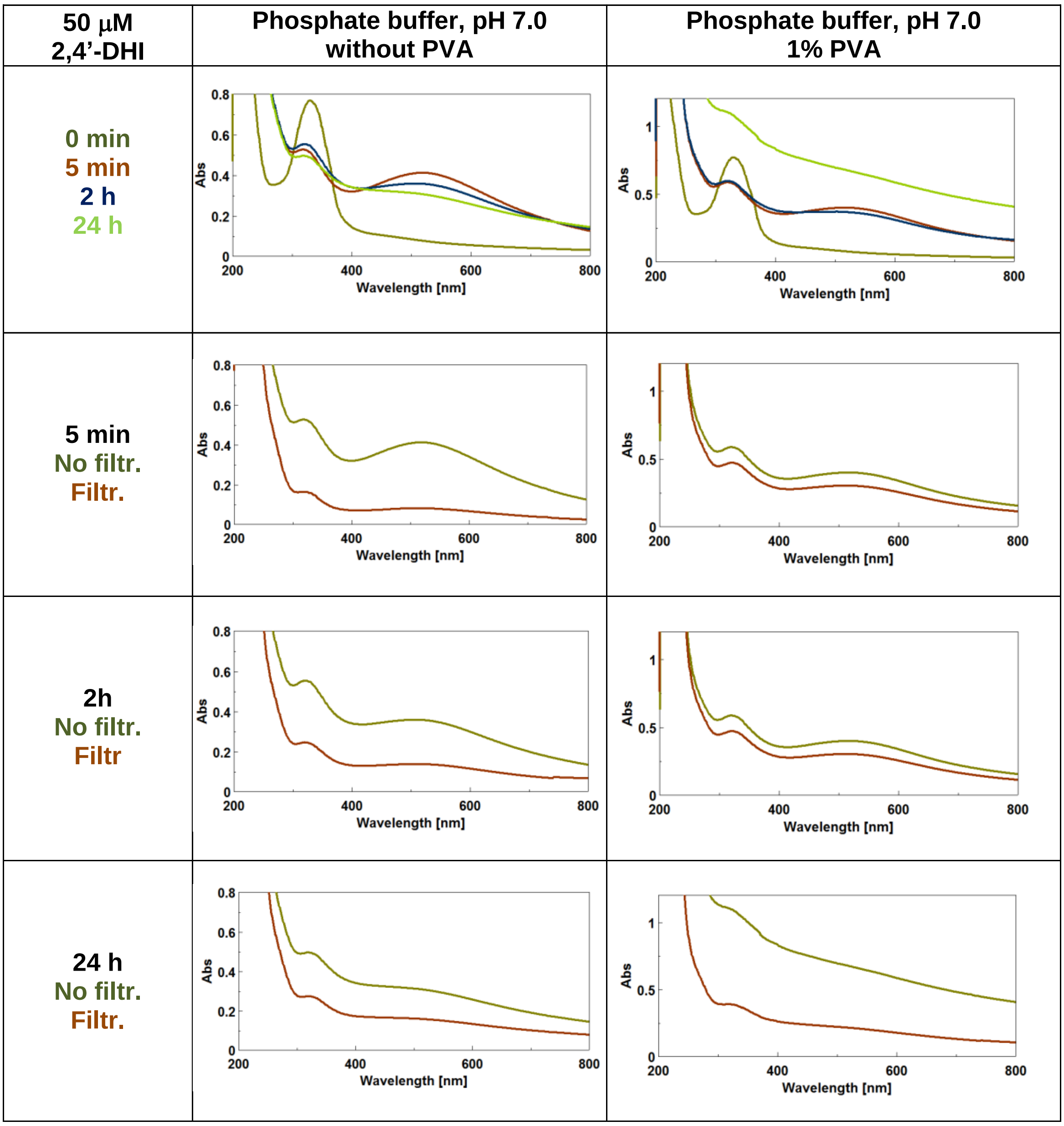

Supplementary Figure 10| Generation and evolution of chromophores from oxidation of 2,4'-DHI dimer by ferricyanide in the absence and in the presence of 1% PVA, with or without filtration.

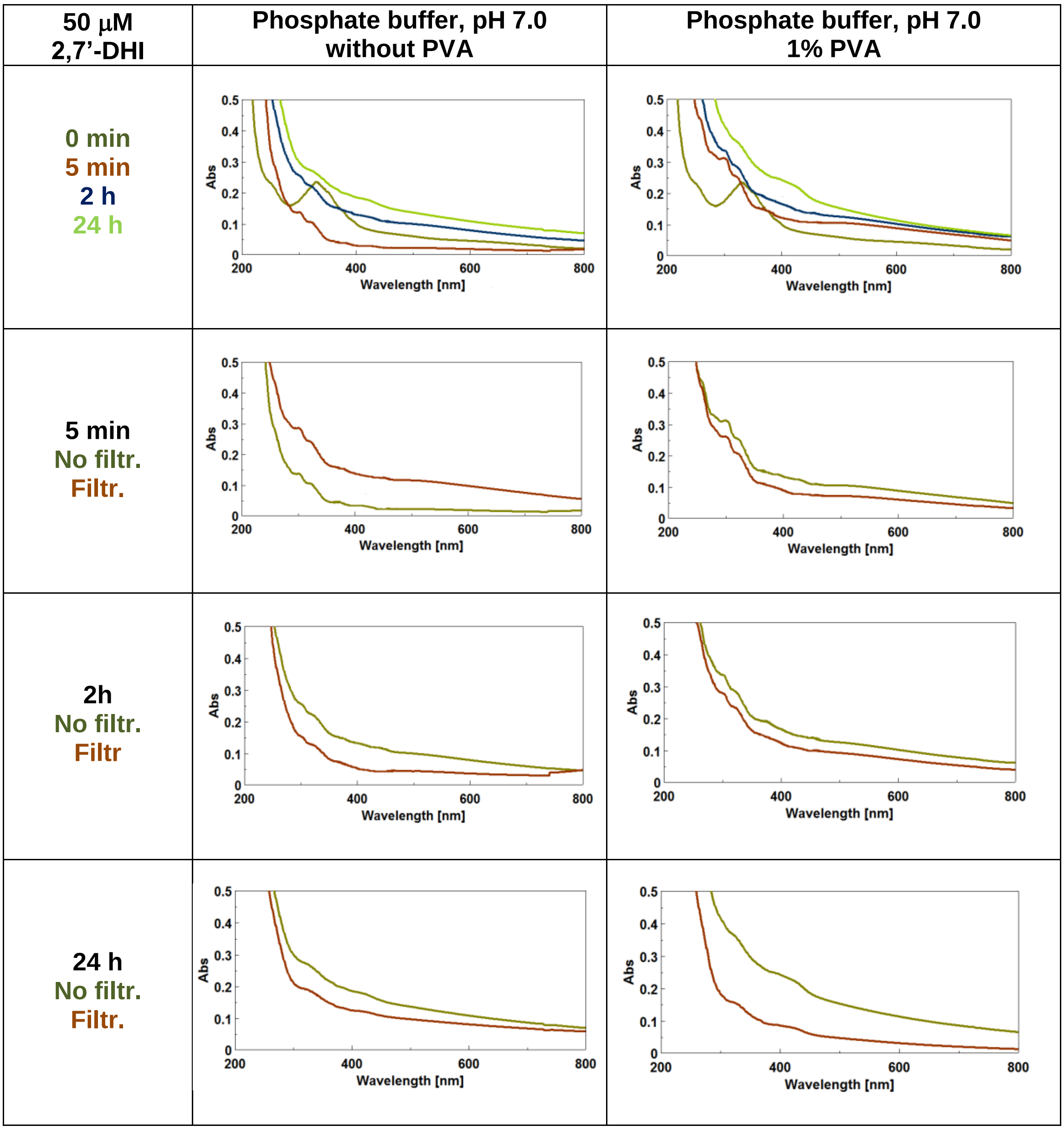

Supplementary Figure 11|Generation and evolution of chromophores from oxidation of 2,7'-DHI dimer by ferricyanide in the absence and in the presence of 1% PVA, with or without filtration.

50 µM<br>4,4’-DHICA	Phosphate buffer, pH 7.0<br>without PVA	Phosphate buffer, pH 7.0<br>with 1% PVA
0 min<br>5 min<br>2 h<br>24 h	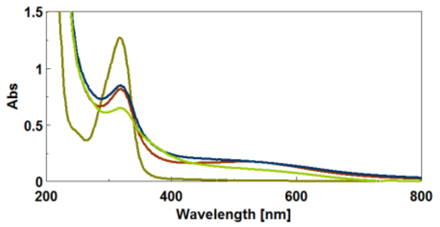	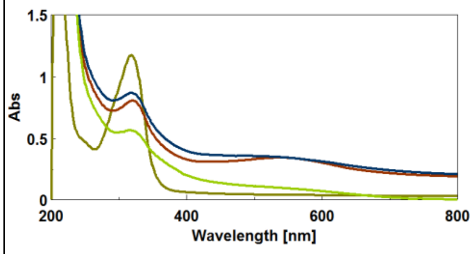
5 min<br>No filtr.<br>Filtr.	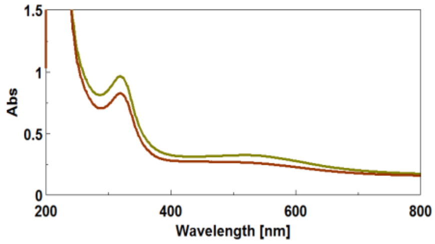	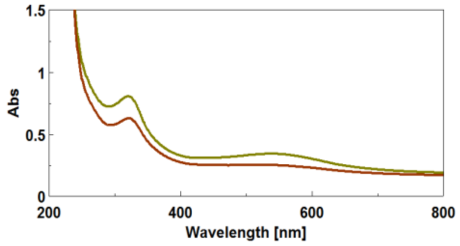
2 h<br>No filtr.<br>Filtr.	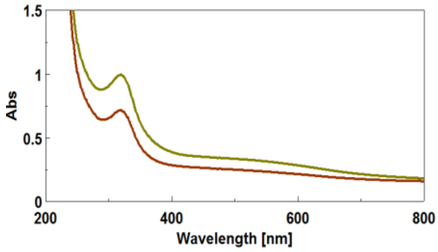	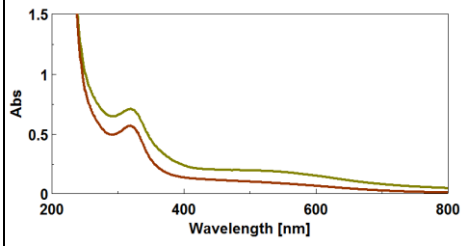
24 h<br>No filtr.<br>Filtr.	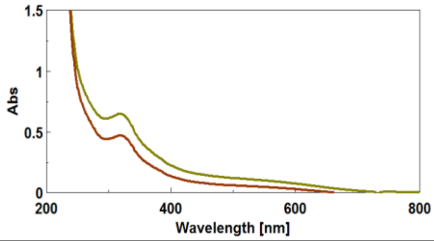	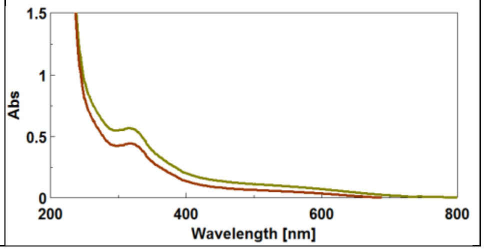

Supplementary Figure 12| Generation and evolution of chromophores from oxidation of 4,4’-DHICA dimer by ferricyanide in the absence and in the presence of 1% PVA, with or without filtration.

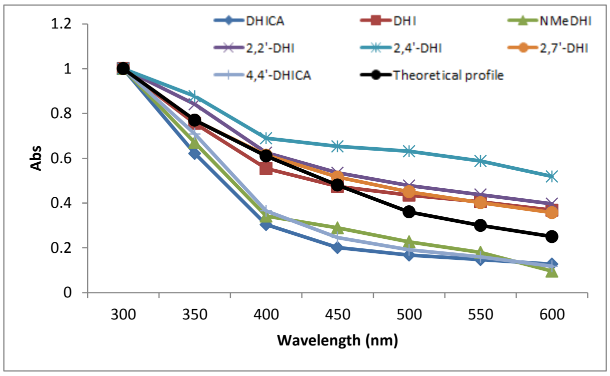

Supplementary Figure 13| Relative absorbance of the of the oxidation mixtures of the indole compounds at 24 h over the UV-visible region in the absence of PVA (values normalized against the absorbance at 300 nm for each compound).

50 µM DOPA	Phosphate buffer, pH 7.0 without PVA	Phosphate buffer, pH 7.0 with 1% PVA
0 min<br>5 min<br>2 h<br>24 h	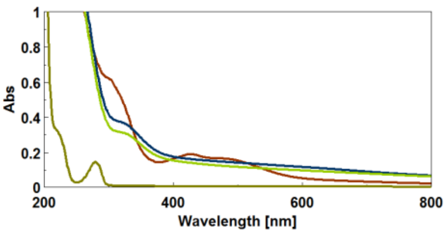	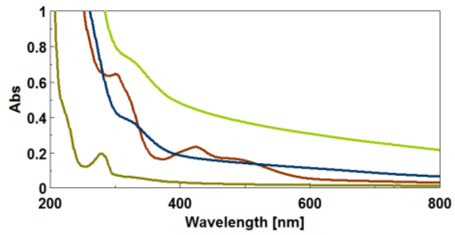
5 min<br>No filtr.<br>Filtr.	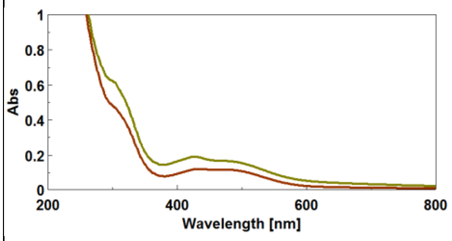	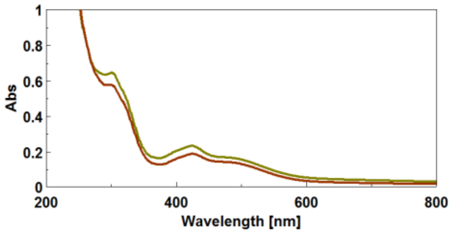
2 h<br>No filtr.<br>Filtr.	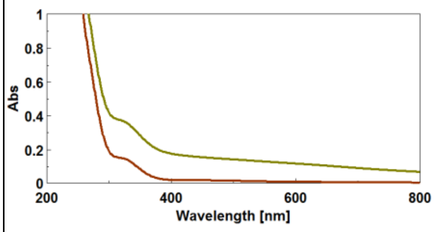	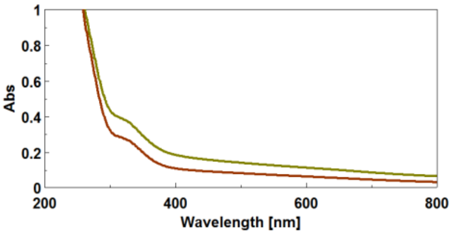
24 h<br>No filtr.<br>Filtr.	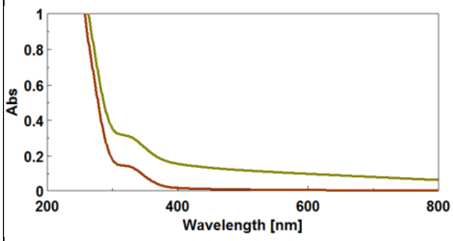	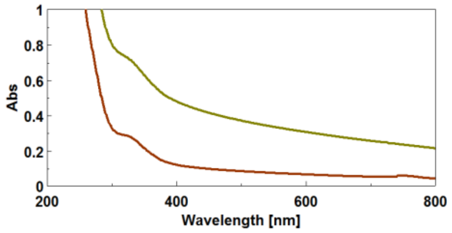

Supplementary Figure 14| UV-vis spectra of oxidation mixture of DOPA by ferricyanide in the absence and in the presence of 1% PVA, with or without filtration. Note that the absorption maximum at around 420 nm observed in the spectra taken at 5 min is due to residual ferricyanide.

50 µM DOPAMINE	Phosphate buffer, pH 7.0 without PVA	Phosphate buffer, pH 7.0 with 1% PVA
0 min<br>5 min<br>2 h<br>24 h	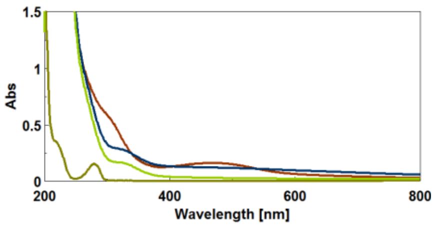	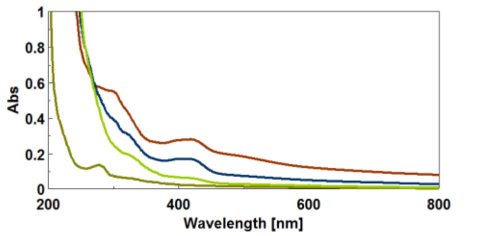
5 min<br>No filtr.<br>Filtr.	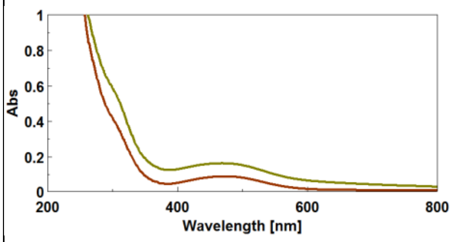	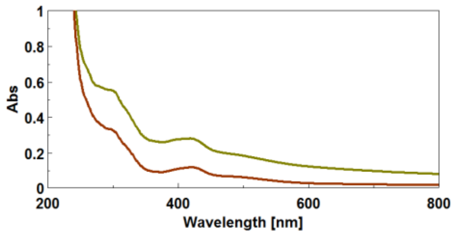
2 h<br>No filtr.<br>Filtr.	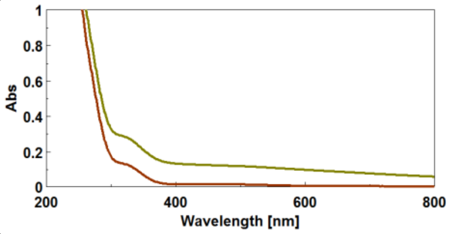	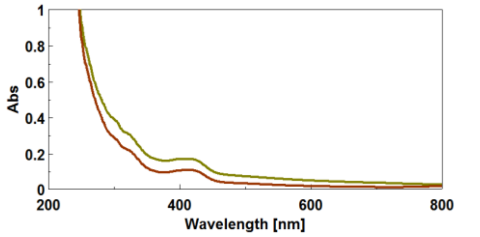
24 h<br>No filtr.<br>Filtr.	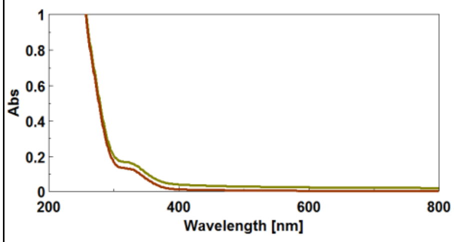	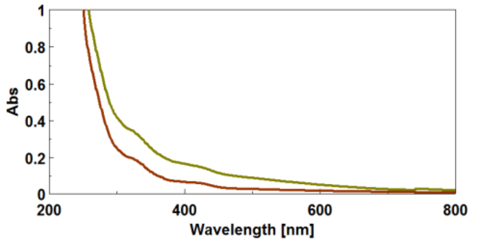

Supplementary Figure 15| UV-vis spectra of oxidation mixture of dopamine by ferricyanide in the absence and in the presence of 1% PVA, with or without filtration.

	DHI, 1% PVA	DHICA, 1% PVA
**0 min**<br>**5 min**<br>**2 eq. $Na_2S_2O_4$**	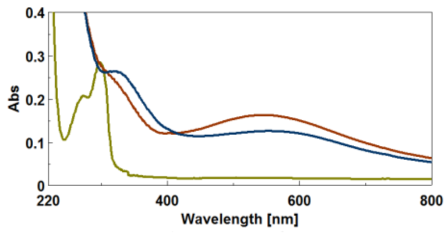	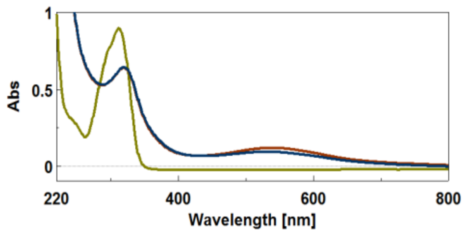
**0 min**<br>**5 min**<br>**2 eq. $NaBH_4$**	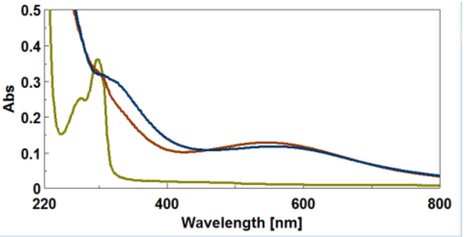	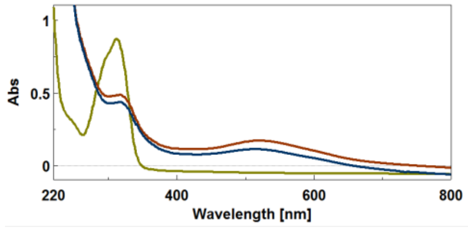

	DHI, without PVA	DHICA, without PVA
**0 min**<br>**5 min**<br>**excess**<br>**$Na_2S_2O_4$**	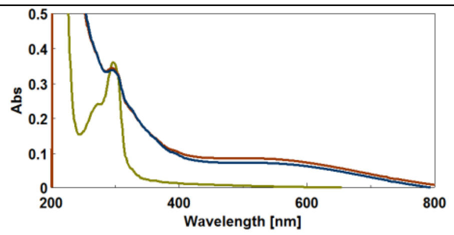	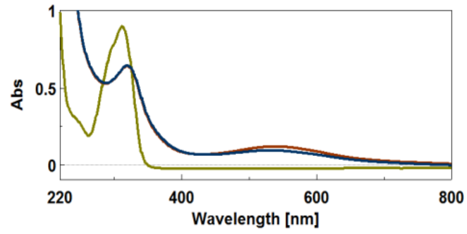
**0 min**<br>**5 min**<br>**excess**<br>**$NaBH_4$**	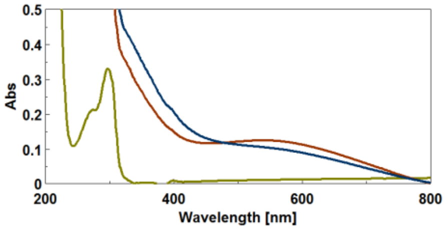	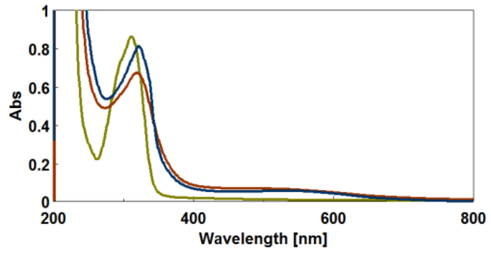

Supplementary Figure 16| Effect of addition of sodium dithionite or sodium borohydride at different molar ratio with respect to the monomer to the oxidation mixtures in the presence or in the absence of 1% PVA.

	DHI	DHICA
0 min<br>5 min<br>Acidification to pH 2	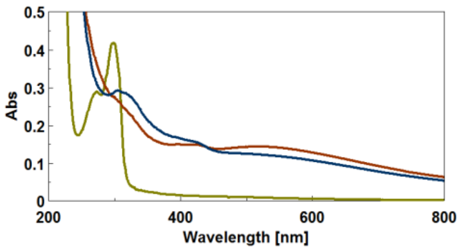	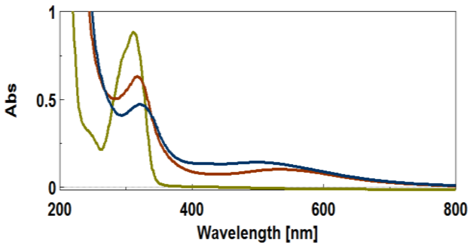

Supplementary Figure 17| Effect of acidification on the chromophores of DHI and DHICA oxidation mixtures.

	DHI	DHICA
a) 50 μM precursor<br><br>b) Addition of 2 eq. $K_3[Fe(CN)_6]$<br><br>c) Addition of 50 μM DHI or DHICA<br><br>d) Sum of spectra (a) and (b)	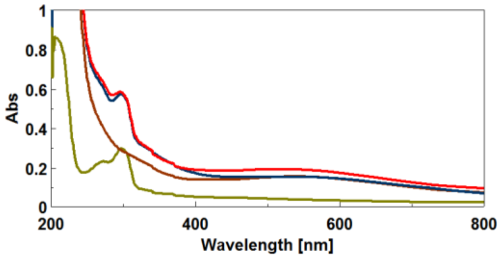	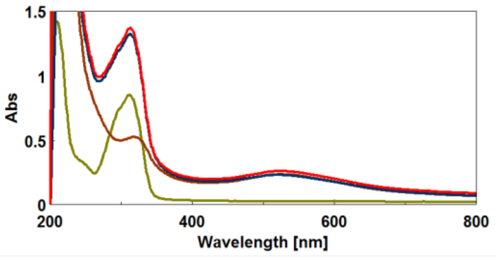

Supplementary Figure 18| Effect of addition of DHI and DHICA at 50 μM to the respective melanin generated in the presence of 1% PVA.

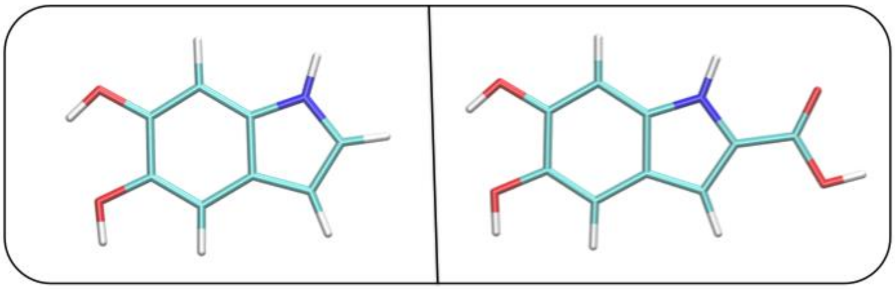

Supplementary Figure 19| DFT Optimized geometries of the investigated monomers: DHI (left) and DHICA (right).

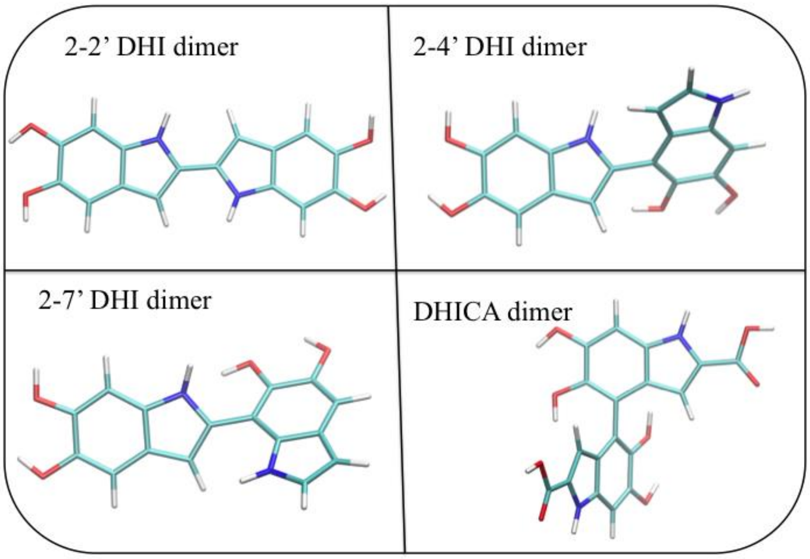

Supplementary Figure 20| DFT Optimized geometries of the investigated dimers in their **HQ** (tetra-hydroxyl) form: 2-2' DHI dimer (top left), 2-4' DHI dimer (top right), 2-7' DHI dimer (bottom left) and DHICA dimer (bottom right). Note the perfectly planar structure of the 2,2' dimer, and the highly distorted scaffold of the DHICA dimer.

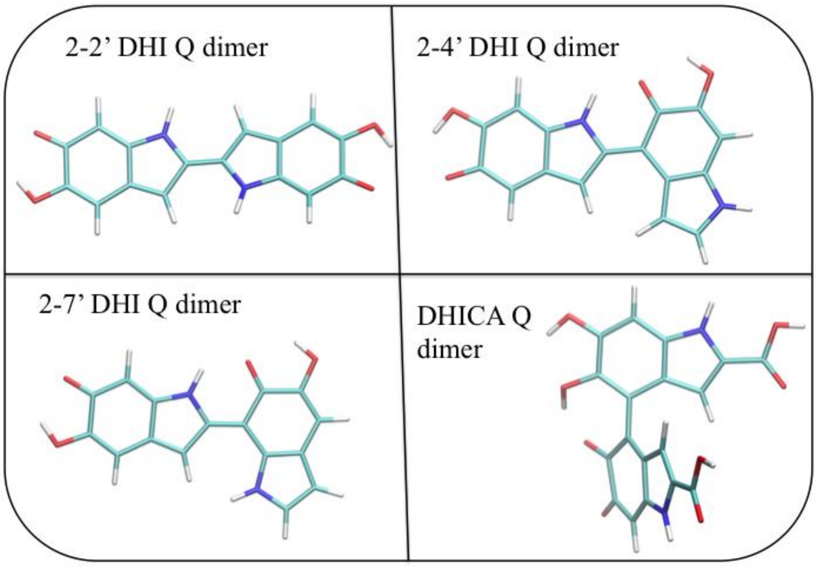

Supplementary Figure 21| DFT Optimized geometries of the investigated dimers in their oxidized **Q** (di-hydroxyl) form: 2-2' DHI dimer (top left), 2-4' DHI dimer (top right), 2-7' DHI dimer (bottom left) and DHICA dimer (bottom right). Note that, upon oxidation, all DHI dimers are now perfectly planar, whereas steric encumbrance still prevents DHICA from planarity.

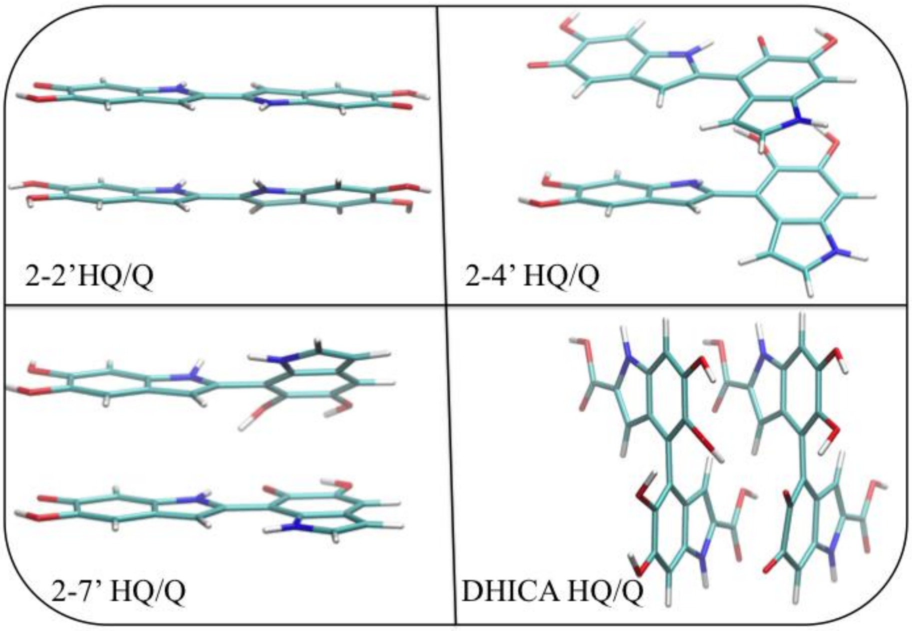

Supplementary Figure 22| Sample geometries of the investigated stacked **HQ**/**Q** dimer pairs (2,2' DHI **HQ**/**Q** pair, top left; 2,4' DHI **HQ**/**Q** pair, top right, 2,7' DHI **HQ**/**Q** pair, bottom left ; 4,4'DHICA **HQ**/**Q** pair, bottom right) employed in the MP2 calculation of interaction energies.

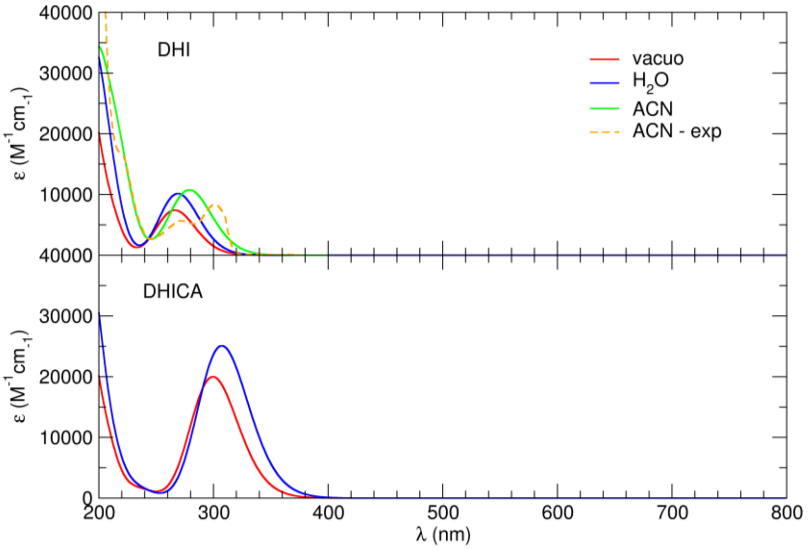

Supplementary Figure 23| Absorption spectra for DHI (top) and DHICA (bottom) monomers, computed at TD-DFT level *in vacuo* (red lines), H_2O (blue) and ACN (green) solvent. Experimental signal (orange dashed line, top panel) recorded for DHI in ACN is also displayed for comparison.[1]

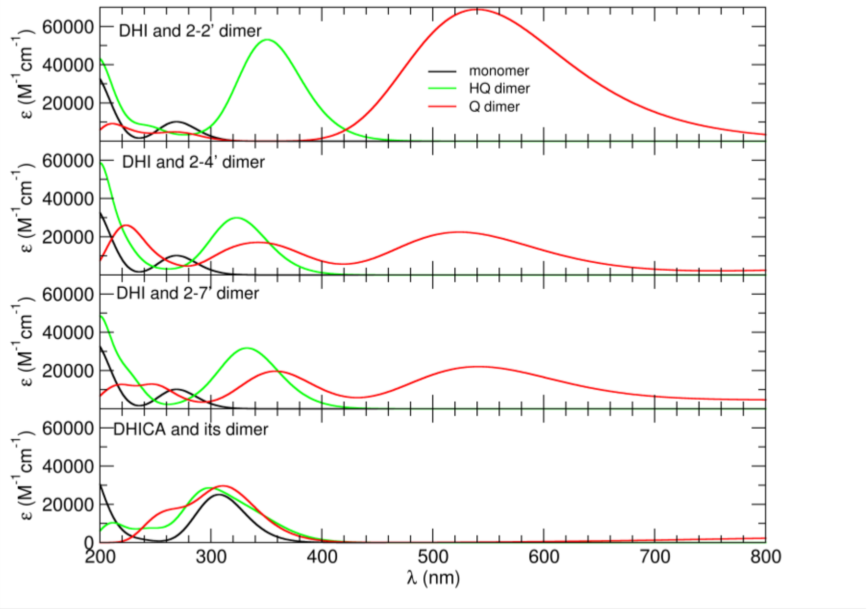

Supplementary Figure 24| Absorption spectra for DHI and DHICA monomers (black lines) and **HQ** (green lines) or **Q** (red lines) dimers computed at TD-DFT level in H_2O.

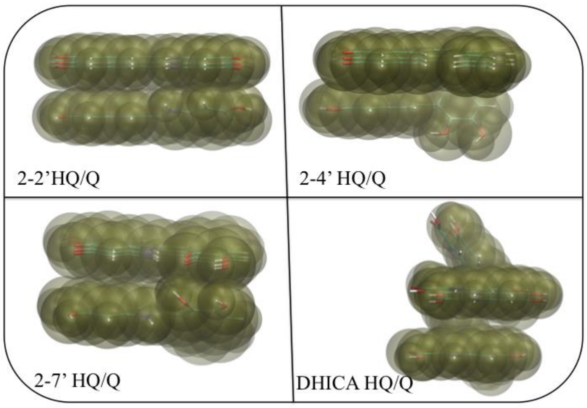

Supplementary Figure 25| Overlap of the van der Waals surfaces upon displacement of the **Q** dimer with respect to the **HQ** dimer in a stacked pair. Displayed conformations correspond to the interaction energies evidenced with red boxes in Figure 6 in the main text. The Q species for each conformer can be spotted inside the upper surface.

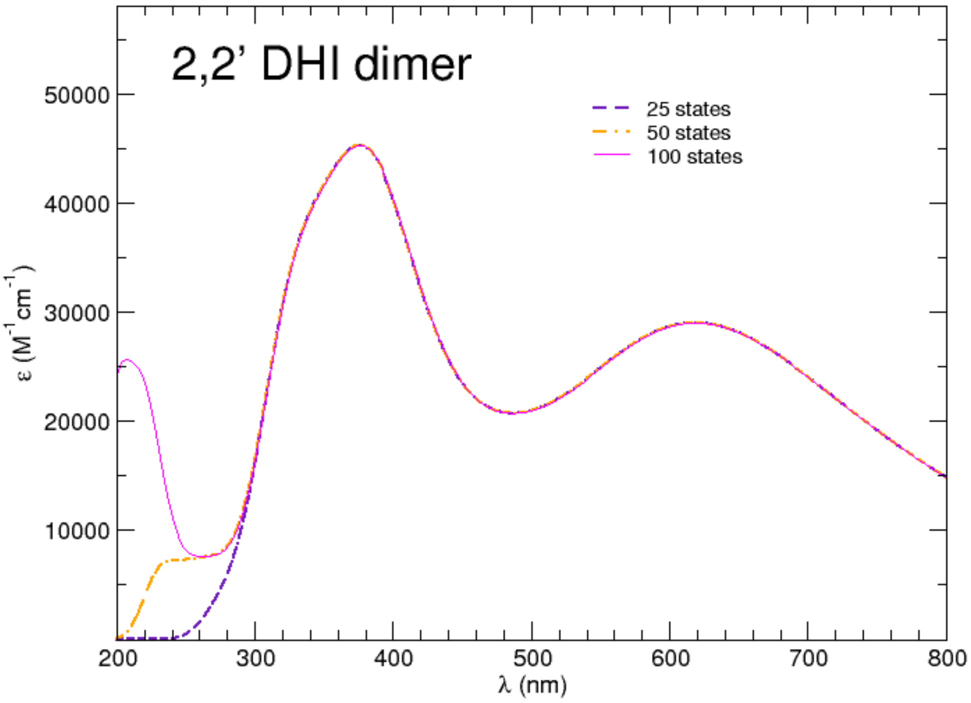

Supplementary Figure 26| Absorption spectra for a pair (HQ/Q) of 2,2' DHI stacked dimers, computed at TD-DFT level *in vacuo* with 25, 50 and 100 excited states. This figure shows that the differences observed at low wave length between the experimental and the spectra computed with 25 excited states are due only to the limited number of eigenstate considered.

References.

1. Zhang, X., Erb, C., Flammer, J. & Nau, W. M. Absolute rate constants for the quenching of reactive excited states by melanin and related 5,6-dihydroxyindole metabolites: implications for their antioxidant activity. *Photochem Photobiol.* **71,** 524-533 (2000).
